# Cannabidiol as the Substrate in Acid-Catalyzed Intramolecular Cyclization

**DOI:** 10.1021/acs.jnatprod.0c00436

**Published:** 2020-09-29

**Authors:** Paola Marzullo, Francesca Foschi, Davide Andrea Coppini, Fabiola Fanchini, Lucia Magnani, Selina Rusconi, Marcello Luzzani, Daniele Passarella

**Affiliations:** Dipartimento di Chimica, Università degli Studi di Milano, Milan 20133, Italy

## Abstract

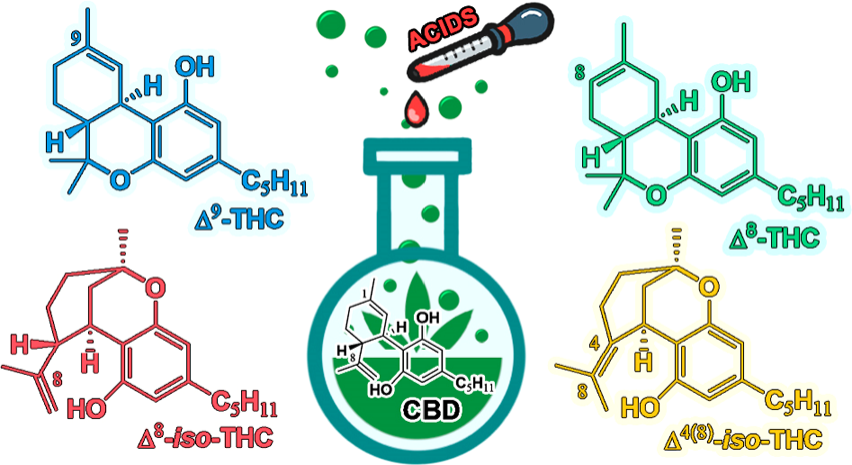

The chemical reactivity of cannabidiol is based on its ability to undergo intramolecular cyclization driven by the addition of a phenolic group to one of its two double bonds. The main products of this cyclization are Δ^9^-THC (*trans*-Δ-9-tetrahydrocannabinol) and Δ^8^-THC (*trans*-Δ-8-tetrahydrocannabinol). These two cannabinoids are isomers, and the first one is a frequently investigated psychoactive compound and pharmaceutical agent. The isomers Δ^8^-*iso*-THC (*trans*-Δ-8-*iso*-tetrahydrocannabinol) and Δ^4(8)^-*iso*-THC (*trans*-Δ-4,8-*iso*-tetrahydrocannabinol) have been identified as additional products of intramolecular cyclization. The use of Lewis and protic acids in different solvents has been studied to investigate the possible modulation of the reactivity of CBD (cannabidiol). The complete NMR spectroscopic characterizations of the four isomers are reported. High-performance liquid chromatography analysis and ^1^H NMR spectra of the reaction mixture were used to assess the percentage ratio of the compounds formed.

Recent years have seen a dramatically increasing interest in phytocannabinoids. Isolated from *Cannabis* in 1940,^1,2^ cannabidiol (CBD) is one of the most abundant phytocannabinoids in the species of *Cannabis* for textile uses.^3,4^ Despite the structural similarity between CBD and Δ^9^-THC (*trans*-Δ-9-tetrahydrocannabinol) (Figure 1), CBD has a low agonistic effect for cannabinoid receptors; in particular, it is considered an allosteric negative modulator of CB1 and CB2 receptors (cannabinoid receptor types 1 and 2).^5,6^ Current evidence shows that CBD exerts pharmacological effects via specific molecular targets such as adenosine, glycine, opioid, serotonin, nonendocannabinoid G protein-coupled, nicotinic acetylcholine, and proliferator-activated receptors.^7^ Moreover, CBD shows anticonvulsant, antispasmodic, anxiolytic, antinausea, antirheumatoid arthritis, and neuroprotective properties.^5^ Recently, it has been demonstrated that CBD is an inverse agonist for G protein-coupled orphan receptors, such as GPR3, GPR6, and GPR12, suggesting new therapeutic uses of CBD for Alzheimer’s disease, Parkinson’s disease, cancer, and infertility.^8^

**Figure 1:**
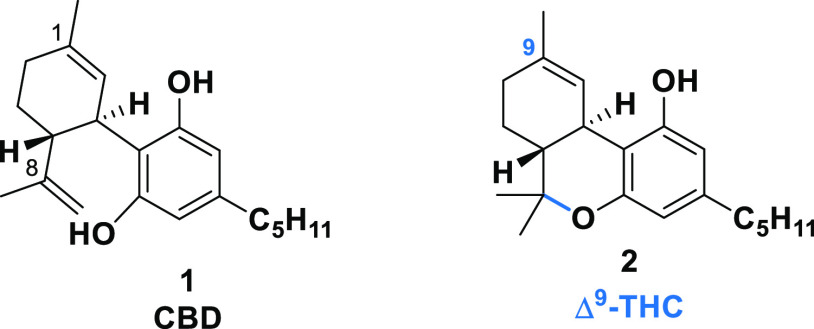
Structures of cannabidiol (CBD) and Δ-9-tetrahydrocannabinol (Δ^9^-THC).

Δ^9^-THC is the key compound of *Cannabis sativa* with major psychoactive effects.^5^ From a pharmacological perspective, Δ^9^-THC is a partial agonist at both cannabinoid receptors: CB1, a modulator of psychoactive effects, and CB2, a modulator of immunological and anti-inflammatory effects.^5^ The psychoactive effects of Δ^9^-THC include anxiety, paranoia, perceptual alterations, and cognitive deficits. All these CB1-mediated effects are caused by the perturbation of GABA (γ-aminobutyric acid)/glutamatergic neurotransmission and dopamine release, and above all, they are generally acute, transient, and self-limited.^5^ Moreover, a low Δ^9^-THC acute toxicity in murine models has also been observed. Lastly, after Δ^9^-THC administration, hypolocomotion, hypothermia, catalepsy, analgesia, and increased food intake have been reported.^5^

The possibility of inducing intramolecular cyclization of CBD to create the THC skeleton is well-known. Because of the remarkable difference in terms of the activity between CBD, Δ^9^-THC, and its isomers, we decided to study (a) the feasibility of this reaction, (b) its selectivity, and (c) the availability of an efficient and quick method for monitoring this conversion.

Thus, CBD was treated with Lewis and protic acids, and the composition of the resulting mixture was evaluated using high-performance liquid chromatography (HPLC) or direct NMR spectra analysis.

## Results and Discussion

**Scheme 1:**
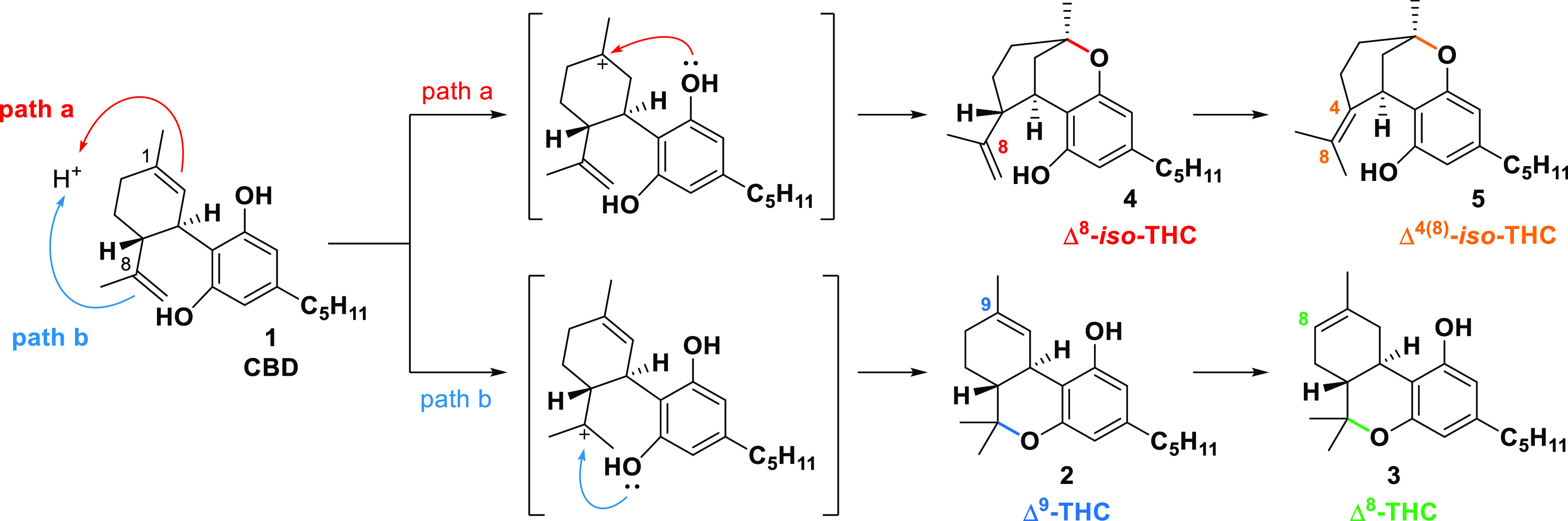
CBD Acid-Promoted Cyclization

According to the literature, the cyclization reaction of CBD seems to occur following an acid-catalyzed activation of a specific double bond.^9,10^ A dihydrobenzopyran ring moiety is formed by internal ether formation of one of the phenolic groups with one of the double bonds. The two double bonds in the CBD structure are responsible for the formation of two different compounds (Scheme 1). If the activation occurs on the Δ^8^ double bond, the products show the THC scaffold (Δ^9^-THC, path b); otherwise, the Δ^1^ double bond activation leads to the formation of the *iso*-THC scaffold (Δ^8^-*iso*-THC, path a). The latter cyclization is much less frequent. However, acidic conditions are responsible for further isomerization toward the corresponding thermodynamically more stable compounds, Δ^8^-THC and Δ^4(8)^-*iso*-THC, respectively.^11^

Although Δ^9^-THC and its derivatives have been widely explored and recognized as the major psychoactive *Cannabis* constituents, the *iso*-THC isomers have received little attention. For this reason, we wish to fill the literature gap, especially regarding the provision of full NMR data.

**Scheme 2:**
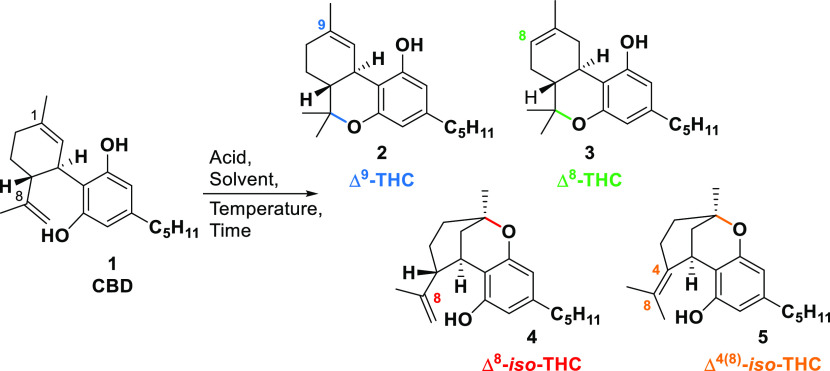
CBD Conversions with Acids and the Structures of the Products

To investigate the susceptibility and selectivity of CBD cyclization, different reactions, including the use of Lewis and protic acids in different solvents and varying the temperature and reaction time, were performed (Scheme 2).

The Lewis acids were evaluated first, starting with the recorded use of BF_3_·OEt_2_.^12,13^ The data suggest that performing the reaction with BF_3_·OEt_2_ in CH_2_Cl_2_ at a low temperature affords Δ^9^-THC as the main product, but increasing the temperature and reaction time results in preferential formation of the more stable Δ^8^-THC. The results support this assertion (Table 1, entries 1 and 2). Lowering the temperature also lowers the yields (Table 1, entry 3). Using other solvents gave different degrees of selectivity. In particular, toluene gave results similar to those in CH_2_Cl_2_, but *iso*-THCs always accompanied the Δ^8^- and Δ^9^-THCs (Table 1, entries 4 and 5). A reaction conducted in MeCN at −10 °C for 6 h yielded Δ^8^-*iso*-THC as the main product accompanied by trace amounts of Δ^4(8)^-*iso*-THC (Table 1, entry 7).

**Table 1:** Reaction Conditions Screening of Acid-Catalyzed Cyclization of CBD Using Lewis Acids^a^

					reaction mixture composition (%)^b^			
entry	acid	solvent	*T* (°C)	time (h)	Δ^9^-THC	Δ^8^-THC	Δ^8^-*iso*-THC	Δ^4(8)^-*iso*-THC
1	BF_3_·OEt_2_	CH_2_Cl_2_	–10	4	44	1	3	
2	BF_3_·OEt_2_	CH_2_Cl_2_	0	6	2	52		
3	BF_3_·OEt_2_	CH_2_Cl_2_	–78 to −30	48	10	11	5	
4	BF_3_·OEt_2_	Tol	–10	3	41	2	29	
5	BF_3_·OEt_2_	Tol	0	6		36		26
6	BF_3_·OEt_2_	THF	–10	6	NR	NR	NR	NR
7	BF_3_·OEt_2_	MeCN	–10	6		5	30	5
8	TMSOTf	CH_2_Cl_2_	–10	6		93		
9	TMSOTf	Tol	–10	6	12	75		
10	In(OTf)_3_	CH_2_Cl_2_	–10	6	52	6	4	
11	In(OTf)_3_	CH_2_Cl_2_	0 to RT	48		72		
12	In(OTf)_3_	Tol	–10	4	NR	NR	NR	NR
13	In(OTf)_3_	Tol	0	24		98		
14	ZnBr_2_	CH_2_Cl_2_	RT	96	NR	NR	NR	NR
15	TiCl_4_	CH_2_Cl_2_	–10	6	34	9		

a RT, room temperature; NR, no reaction.

b Determined via HPLC and ^1^H NMR analysis.

To enhance the yields and the selectivity of the process, a series of tests with different acids were conducted, following the hypothesis that other Lewis acids could actively induce cyclization. Starting from the positive literature results regarding the use of TMSI (trimethylsilyl iodide), which showed a high yield of Δ^9^-THC formation without isomerization,^14^ TMSOTf (trimethylsilyl triflate) was tested as an acidic reagent. Contrary to the expectations, it displayed a high affinity for the formation of Δ^8^-THC when CH_2_Cl_2_ or toluene were used as solvents, even at a low temperature (Table 1, entries 8 and 9).

In(OTf)_3_ in CH_2_Cl_2_ converted CBD into Δ^9^-THC at a low temperature in a better yield than that of BF_3_·OEt_2_ (Table 1, entry 10). As in previous tests, higher temperatures caused the production of the thermodynamically more stable isomer in higher yields (Table 1, entry 11). Using toluene, the selectivity shifted to the formation of Δ^8^-THC in excellent yields (Table 1, entry 13). The use of ZnBr_2_ in CH_2_Cl_2_ did not promote the cyclization reaction even at room temperature (Table 1, entry 14), while TiCl_4_ showed a trend similar to that of BF_3_ (Table 1, entry 15). The activity of AlCl_3_, AgOTf, and Ti(OiPr)_4_ was also investigated, without any noteworthy results. Considering these outcomes, a unique preferential formation path for any of the possible isomers cannot be determined based on the characteristics of the Lewis acid used to induce the cyclization.

Subsequently, protic acid screening was performed (Table 2). The best results for CBD conversion were obtained with HCl, pTSA (*p-*toluenesulfonic acid), and CSA (camphorsulfonic acid). As reported,^9^ pTSA in CH_2_Cl_2_ led directly to the formation of Δ^8^-THC as the sole product (Table 2, entry 2). The nature of the solvent clearly affected the reaction outcome. The reaction in *n-*hexane afforded a mixture of Δ^9^-THC, Δ^8^-THC, and Δ^8^-*iso*-THC in a ratio of 1:5:1 (Table 2, entry 3), while the reaction in toluene gave a higher selectivity. pTSA gave different isomers in different percentages depending on the solvent and reaction time (Table 2, entries 2–5). The best selectivity of Δ^9^-THC and Δ^8^-THC formation was obtained with toluene and CH_2_Cl_2_, respectively. On the contrary, the use of 10% mmol catalytic amounts of acid in toluene resulted in almost complete isomerization of the double bond because of the increased reaction time that shifted the outcome to the thermodynamic isomer (Table 2, entry 6). Interestingly, CSA promoted the cyclization of CBD to Δ^9^-THC with complete selectivity and satisfactory yields regardless of the reaction time (Table 2, entry 7). Other protic acids gave worse results for the CBD conversion (Table 2, entries 8–15).

**Table 2:** Reaction Conditions Screening of Acid-Catalyzed Cyclization of CBD Using Protic Acids^a^

					reaction mixture composition (%)^b^			
entry	acid	solvent	*T* (°C)	time (h)	Δ^9^-THC	Δ^8^-THC	Δ^8^-*iso*-THC	Δ^4(8)^-*iso*-THC
1	HCl	H_2_O	RT	72		57		
2	pTSA	CH_2_Cl_2_	RT	36		94		
3	pTSA	*n*-Hex	RT	36	13	66	13	
4	pTSA	DMSO	RT	18	NR			
5	pTSA	Tol	RT	48	82	11		
6	pTSA cat^c^	Tol	RT	96	9	89		
7	CSA	Tol	RT	96	61			
8	H_2_SO_4_	CH_2_Cl_2_	0	72		5	4	11
9	H_2_SO_4_	Tol	RT	96	NR	NR	NR	NR
10	ascorbic acid	CH_2_Cl_2_	0	24	NR	NR	NR	NR
11	ascorbic acid	Tol	RT	96	NR	NR	NR	NR
12	citric acid	EtOH	RT	96	NR	NR	NR	NR
13	HOAc	CH_2_Cl_2_	0	24	NR	NR	NR	NR
14	HOAc	Tol	RT	96	NR	NR	NR	NR
15	H_3_PO_4_	Tol	–10 to 50	48	NR	NR	NR	NR

a RT, room temperature; NR, no reaction.

b Determined via HPLC and ^1^H NMR analysis.

c pTSA 10% catalytic amount.

Some Δ^4(8)^-*iso*-THC formation was detected in three cases (Table 1, entries 5 and 7; Table 2, entry 8), and this compound was isolated and characterized.

The experimental results indicate that toluene is the most suitable solvent for the conversion of CBD into THC isomers. This solvent particularly affects the selectivity of the isomers according to the other experimental conditions (the reaction temperature and nature of the acid cyclization promoter). Increasing the temperature reduces the selectivity of the activation of the double bond and favors the formation of the corresponding most stable isomers. The Lewis acids BF_3_·OEt_2_, In(OTf)_3_, and TMSOTf have a proven effect and affected the major formation of Δ^8^-THC and the product mixtures. As for protic acids, pTSA promotes the reaction to selectively afford Δ^9^- and Δ^8^-THCs, depending on the reaction time. CSA emerges as an interesting cyclization inducer, giving Δ^9^-THC in good yields through readily accessible reaction conditions.

With these encouraging screening results, the influence of CSA was more thoroughly investigated; therefore, the solvent, temperature, and time were considered variables. Using toluene as the solvent at room temperature gave a 61% yield of Δ^9^-THC accompanied by unreacted CBD (Table 2, entry 7). A longer reaction time led to isomerization of Δ^9^-THC and to decomposition of compounds (Table 3, entry 3). Increasing the temperature led to the formation of a mixture that was enriched with the Δ^8^ isomer over time (Table 3, entries 4–8). Increasing the temperature further drastically reduced the CBD conversion time and isomerization, obtaining Δ^9^-THC as a kinetic reaction product (Table 3, entries 9 and 10). In CH_2_Cl_2_, the reaction was faster and less selective toward Δ^9^-THC formation (Table 3, entries 11–13). *n*-Hexane and MTBE (*t*-butyl methyl ether) induced a high degree of CBD conversion without a marked preferential selectivity for the formation of a THC isomer even at a short reaction time (Table 3, entries 14 and 15). In all experiments, *iso*-THC isomers were not detected except when the reaction was performed in MTBE (Table 3, entry 15).

**Table 3:** Reaction Conditions of the Acid-Catalyzed Cyclization of CBD Using CSA^a^

				reaction mixture composition (%)^b^		
entry	solvent	*T* (°C)	time (h)	Δ^9^-THC	Δ^8^-THC	Δ^8^-*iso*-THC
1	Tol	RT	48			
2	Tol	RT	96	61		
3	Tol	RT	120	20	28	
4	Tol	30	96	53	20	
5	Tol	40	24	48	19	
6	Tol	40	48	45	52	
7	Tol	40	72	28	72	
8	Tol	40	96	13	87	
9	Tol	50	3	37	10	
10	Tol	50	4	62	19	
11	CH_2_Cl_2_	RT	24	33	5	
12	CH_2_Cl_2_	RT	48	64	36	
13	CH_2_Cl_2_	30	24	48	52	
14	*n*-Hex	30	96	31	41	
15	MTBE	30	96	54	26	9
16	cyclohexane	30	96	NR	NR	NR

a RT, room temperature; NR, no reaction.

b Determined via HPLC and ^1^H NMR analysis.

Toluene showed the best selectivity; however, the long reaction time seemed to be a drawback. CH_2_Cl_2_ appeared to be promising in this respect, but continuous monitoring of the reaction was required to avoid the prevalence of isomerization.

Δ^9^-THC, Δ^8^-THC, Δ^8^-*iso*-THC, and Δ^4(8)^-*iso*-THC were fully characterized using NMR data, and the complete **^1^**H and **^13^**C NMR assignments (Table 4–6) have been determined on the basis of 1D and 2D NMR spectra (^1^H and ^13^C NMR, correlation spectroscopy (COSY), heteronuclear single quantum coherence (HSQC), and heteronuclear multiple bond correlation (HMBC)). The data were compared with those available in the literature.^15,16^

**Table 4:**
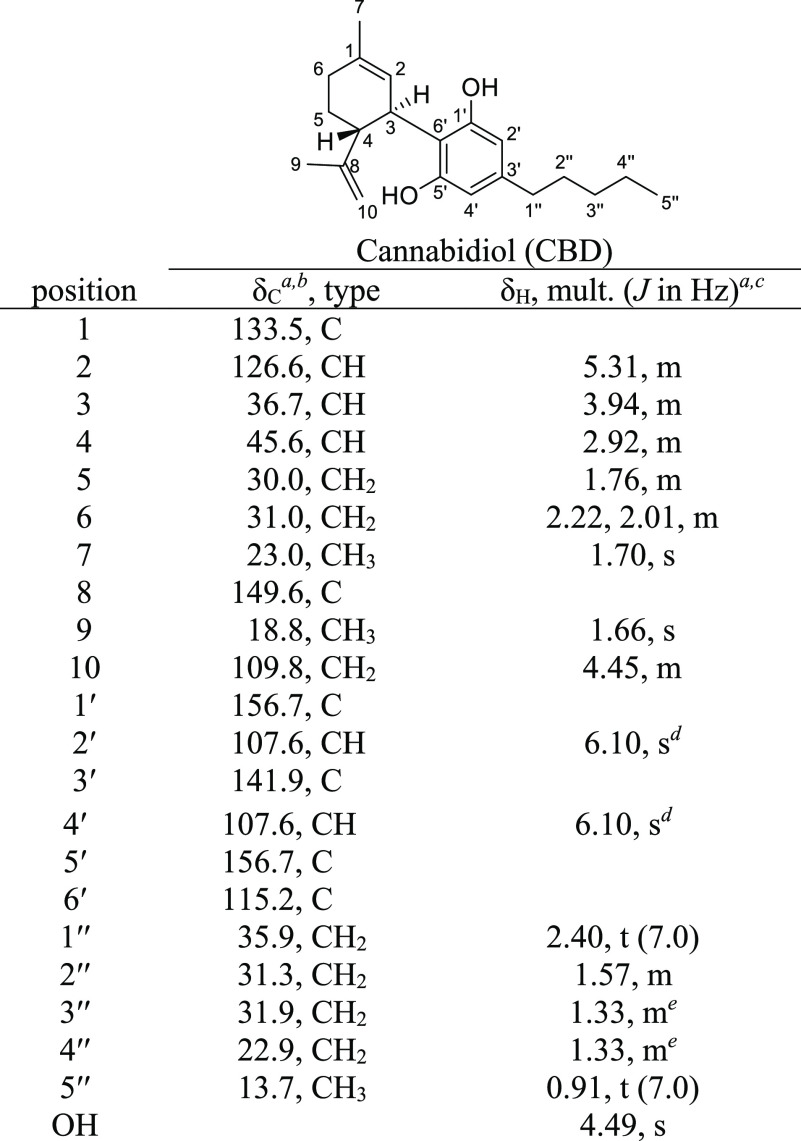
NMR Spectroscopy Data (400 MHz, Methanol-*d*_4_) of CBD

a Chemical shifts (in ppm) were determined with reference to TMS.

b Spectra recorded at 101 MHz.

c Spectra recorded at 400 MHz.

d–e Chemical shifts bearing the same symbol overlap.

**Figure 2:**
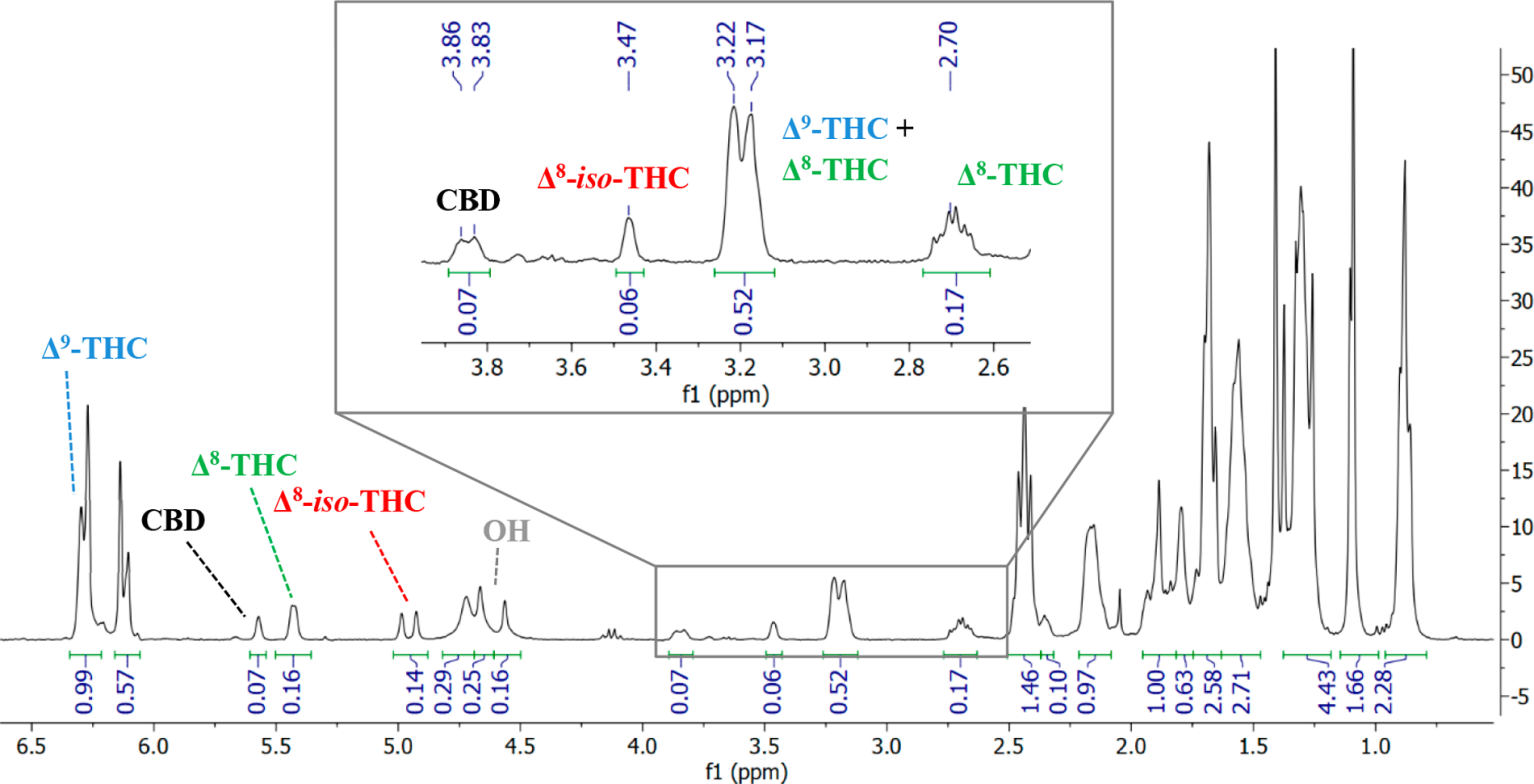
^1^H NMR spectrum in CDCl_3_ of a mixture of CBD, Δ^9^-THC, Δ^8^-THC, and Δ^8^-*iso*-THC.

Diagnostic and distinguishable NMR peaks permit identification of compounds derived from intramolecular cyclization within the crude reaction mixture and also allow the composition percentage to be determined from integration ratios (Figure 2). In CDCl_3_, Δ^9^-THC is characterized by the presence of signals at 6.34 ppm (H-10), 3.23 ppm (H-10a), 2.22–2.16 ppm (H-8), and 4.88 ppm (OH). The ^1^H NMR data of Δ^8^-THC show two signals for H-10 (3.21 and 2.19–2.15 ppm), while the signal due to H-10a is present at 2.71 ppm. The olefinic (H-8) and the hydroxy proton appear at 5.45 and 4.63 ppm, respectively. For Δ^8^-*iso*-THC, the corresponding characteristic signals are the doublet at 4.98 ppm (H-9) and the two signals at 3.49 and 2.37 ppm that match the protons H-3 and H-4, respectively. The spectrum of Δ^4(8)^-*iso*-THC shows a characteristic signal at 4.29 ppm (H-3).

On the basis of the literature data,^17^ an HPLC method was used to follow the cyclization of CBD. The reactions monitored via HPLC provided a composition of the reaction mixture comparable with that of the ^1^H NMR data analysis.

**Figure 3:**
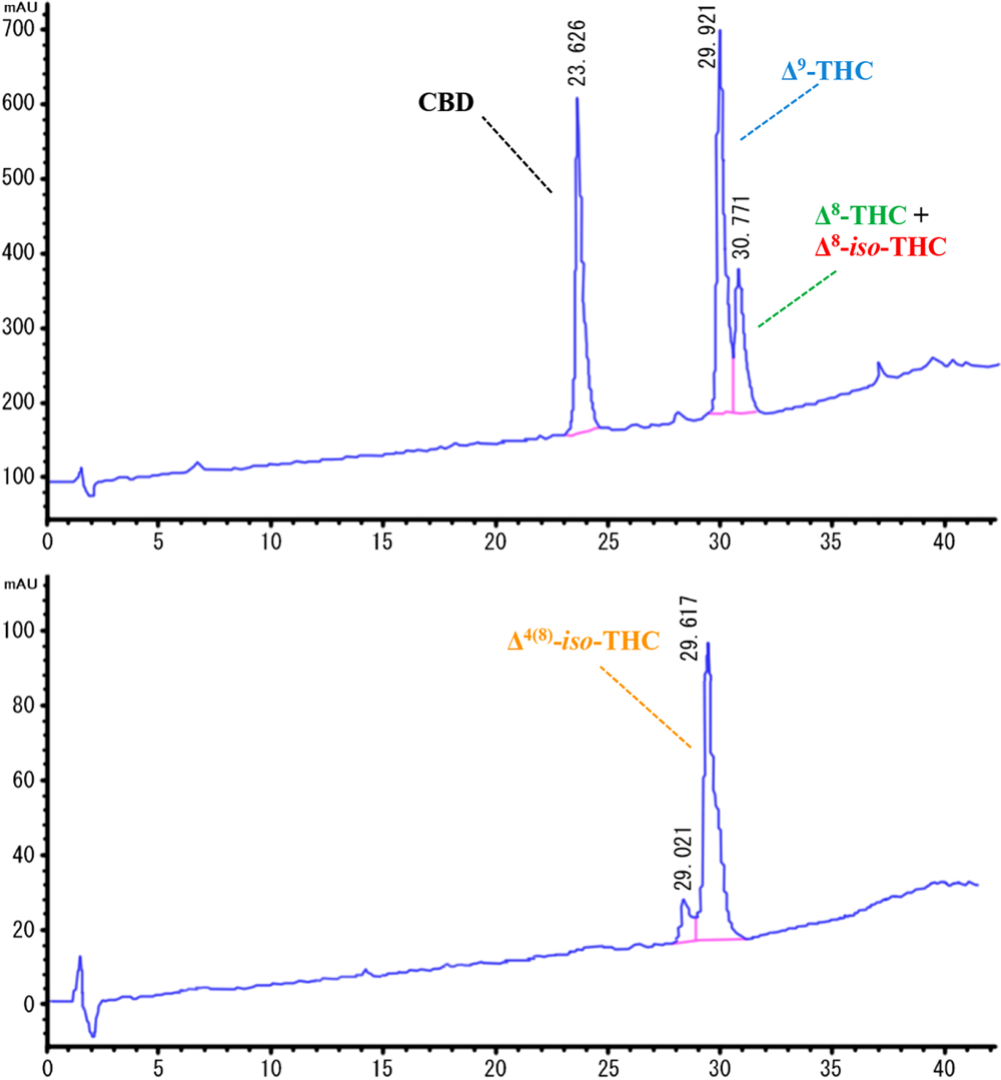
Representative chromatogram of the standard cannabinoid mixture.

The analysis was performed on an ASCENTIS RP-C_18_ column (5 μm × 4.6 × 150 mm). The pressure was set at 101 bar, and the temperature was maintained at 40 °C with a constant flow rate of 0.95 mL/min. UV spectra were recorded at 228.8 nm using a gradient elution method. The mobile phase consisted of a mixture of A (0.1% v/v HCOOH in H_2_O) and B (0.1% v/v HCOOH in MeCN). The gradient elution program was adapted to a 30 min duration to obtain RRT 1.00 for CBD and RRT 1.28 for Δ^9^-THC. After 30 min, the column was purged with 100% B in 7 min; subsequently, the system was washed under these conditions for 3 min and restored to the initial conditions. The retention times were CBD, 23.63 min; Δ^4(8)^-*iso*-THC, 29.62 min; Δ^9^-THC, 29.92 min; Δ^8^-THC, 30.77 min; and Δ^8^-*iso*-THC, 30.77 min (Figure 3).

The method allowed an excellent separation of CBD from the THC isomers; in particular, it was possible to recognize Δ^9^-THC from Δ^8^-THC and Δ^8^-*iso*-THC; however, the peaks were quite close. The drawback was that it remained difficult to obtain a better resolution between the peaks of Δ^9^-THC/Δ^4(8)^-*iso*-THC, and Δ^8^-THC/Δ^8^-*iso*-THC, which have similar retention times. For this reason, the HPLC results were always compared with those obtained from the ^1^H NMR data.

In conclusion, all THC isomers were fully characterized via ^1^H and ^13^C NMR spectroscopy. An analytical method was optimized to monitor the course of the reactions. In particular, it was found that CSA in toluene at room temperature (RT) for 96 h and pTSA in toluene for 48 h at RT were the best conditions for the selective formation of Δ^9^-THC. TMSOTf in CH_2_Cl_2_ at −10 °C for 6 h, In(OTf)_3_ in toluene at 0 °C for 24 h, pTSA in CH_2_Cl_2_ at RT for 36 h, and CSA in toluene at 40 °C for 96 h selectively afforded Δ^8^-THC in high yields. The use of BF_3_·OEt_2_ in toluene led to the formation of the *iso*-THC isomer, depending on the reaction temperature. At −10 °C, a separable mixture of Δ^9^-THC and Δ^8^-*iso*-THC was obtained, whereas a temperature increase to 0 °C shifted the result toward the corresponding most stable isomers, Δ^8^-THC and Δ^4(8)^-*iso*-THC. CBD is a challenging substrate that permits the chemical reactivity of natural alkenes and phenols to be addressed and exploited.

## Experimental Section

### General Experimental Procedures

Unless otherwise stated, reagents and solvents were purchased from Sigma-Aldrich (Milan, Italy), Fluorochem (Hadfield, United Kingdom), or TCI (Zwijndrecht, Belgium) and used without further purification. All reactions were carried out in oven-dried glassware and dry solvents under a nitrogen atmosphere and were monitored by TLC on silica gel (Merck precoated 60F254 plates), with detection by UV light (254 nm) or by cerium molybdate stain (Hanessian’s stain). Analytical HPLC was performed on an ASCENTIS RP-C_18_ column (5 μm × 4.6 × 150 mm). The pressure was set at about 101 bar, and the temperature was maintained at 40 °C, with a constant flow rate of 0.95 mL/min. UV spectra were recorded at 228 nm using a gradient elution method. The mobile phase consisted of a mixture of A (0.1% v/v HCOOH in H_2_O) and B (0.1% v/v HCOOH in MeOH). The gradient was programmed linearly from 60% B to 90% B in 30 min. Flash column chromatography (FCC) was performed using silica gel (240–400 mesh, Merck) as a stationary phase. ^1^H NMR spectra were recorded on a Bruker Avance Spectrometer 300 or 400 MHz, and chemical shifts are reported relative to residual CDCl_3_, methanol-*d*_4_, or acetone-*d*_6_. ^13^C NMR spectra were recorded on the same instrument (101 MHz), and chemical shifts are reported relative to residual CDCl_3_, methanol-*d*_4_, or acetone-*d*_6_. All 1D and 2D NMR spectra were acquired using the standard pulse sequences available with Bruker Topspin 1.3. Chemical shifts (δ) for proton and carbon resonances are quoted in parts per million (ppm) relative to TMS, used as an internal standard. Data for ^1^H NMR are reported as follows: chemical shift (δ/ppm), multiplicity, and coupling constants (Hz). Multiplicities are reported as follows: s = singlet, d = doublet, t = triplet, m = multiplet, and br s= broad singlet. Data for ^13^C NMR are reported in terms of chemical shifts (δ/ppm). MS spectra were recorded using the Electrospray Ionization (ESI) technique on a Waters Micromass quadrupole time-of-flight micro-mass spectrometer, and HR-ESI mass spectra were recorded on a FT-ICR APEXII instrument (Bruker Daltonics). EI mass spectra were recorded at an ionizing voltage of 6 kEv on a VG 70–70 EQ. Optical rotation values were measured on a Jasco P-1030 polarimeter at 20 °C, using a sodium D line wavelength λ = 589 nm.

### General Procedure Using Lewis or Protic Acids

All the reactions were performed under a nitrogen atmosphere in different anhydrous solvents and at different temperatures. To a CBD stirred solution at the specified temperature (more details follow below) was slowly added the corresponding Lewis or protic acids, and the mixture was stirred. The reaction was quenched with a saturated aqueous NaHCO_3_ solution, stirred for 30 min, and washed with a saturated aqueous NaHCO_3_ solution and with brine. The organic phase was dried over Na_2_SO_4_, filtered, and evaporated under reduced pressure. All the reactions were monitored by TLC (CH_2_Cl_2_/*n*-hexane 1:3) developed by cerium molybdate stain, and the crudes were analyzed by ^1^H NMR spectroscopy in CDCl_3_ and HPLC to determine composition. All the residues were purified by FCC on silica gel (CH_2_Cl_2_/*n*-hexane 1:3), providing four possible THC isomers.

#### CBD

[α]_D_^20^ −113 (*c* 1, EtOH); [HPLC ASCENTIS C_18_; RT CBD = 23.63 min]; ^1^H and ^13^C NMR data see Table 4; HRMS (ESI) *m*/*z* [M + Na]^+^ 337.2137 (calcd. for C_21_H_30_O_2_Na, 337.213).

#### Δ^9^-THC

[α]_D_^20^ −159 (*c* 1, CHCl_3_); [HPLC ASCENTIS C_18_; RT Δ^9^-THC = 29.92 min]; ^1^H and ^13^C NMR data see Table 5; HRMS (ESI) *m*/*z* [M + Na]^+^ 337.2132 (calcd for C_21_H_30_O_2_Na, 337.2138).

**Table 5:**
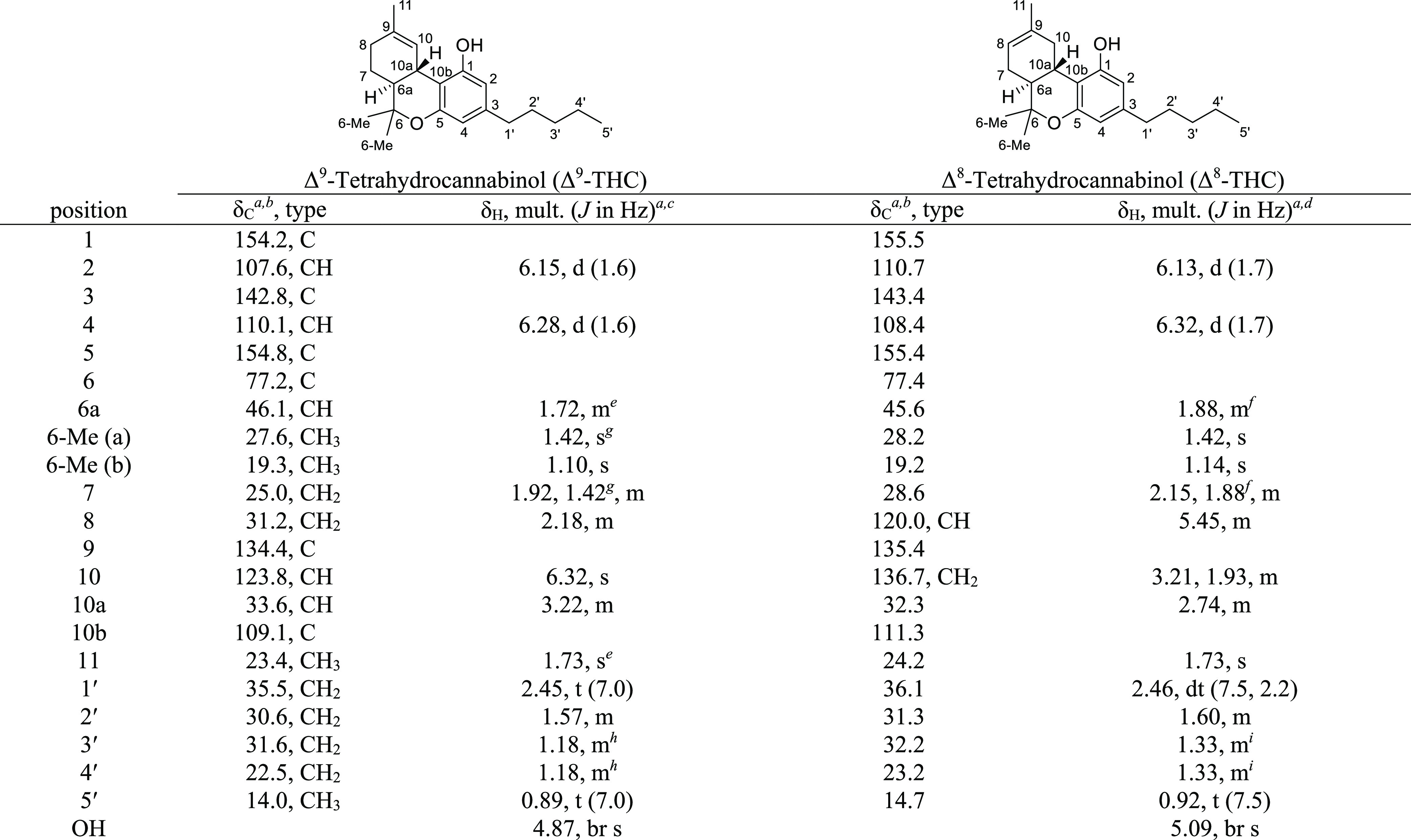
NMR Spectroscopy Data (400 MHz, CDCl_3_) of Δ^9^-THC and (300 MHz, CDCl_3_) of Δ^8^-THC

a Chemical shifts (in ppm) were determined with reference to TMS.

b Spectra recorded at 101 MHz.

c Spectra recorded at 400 MHz.

d Spectra recorded at 300 MHz.

e–i Chemical shifts bearing the same symbol overlap.

#### Δ^8^-THC

[α]_D_^20^ −238 (*c* 1, CHCl_3_); [HPLC ASCENTIS C_18_; RT Δ^8^-THC = 30.77 min]; ^1^H and ^13^C NMR data see Table 5; HRMS (ESI) *m*/*z* [M + Na]^+^ 337.2136 (calcd. for C_21_H_30_O_2_Na, 337.2138).

#### Δ^8^-*iso*-THC

[α]_D_^20^ −249 (*c* 1, CHCl_3_); [HPLC ASCENTIS C_18_; RT Δ^8^*-iso*-THC = 30.77 min]; ^1^H and ^13^C NMR data see Table 6; HRMS (ESI) *m*/*z* [M + Na]^+^ 337.2141 (calcd. for C_21_H_30_O_2_Na, 337.2138).

**Table 6:**
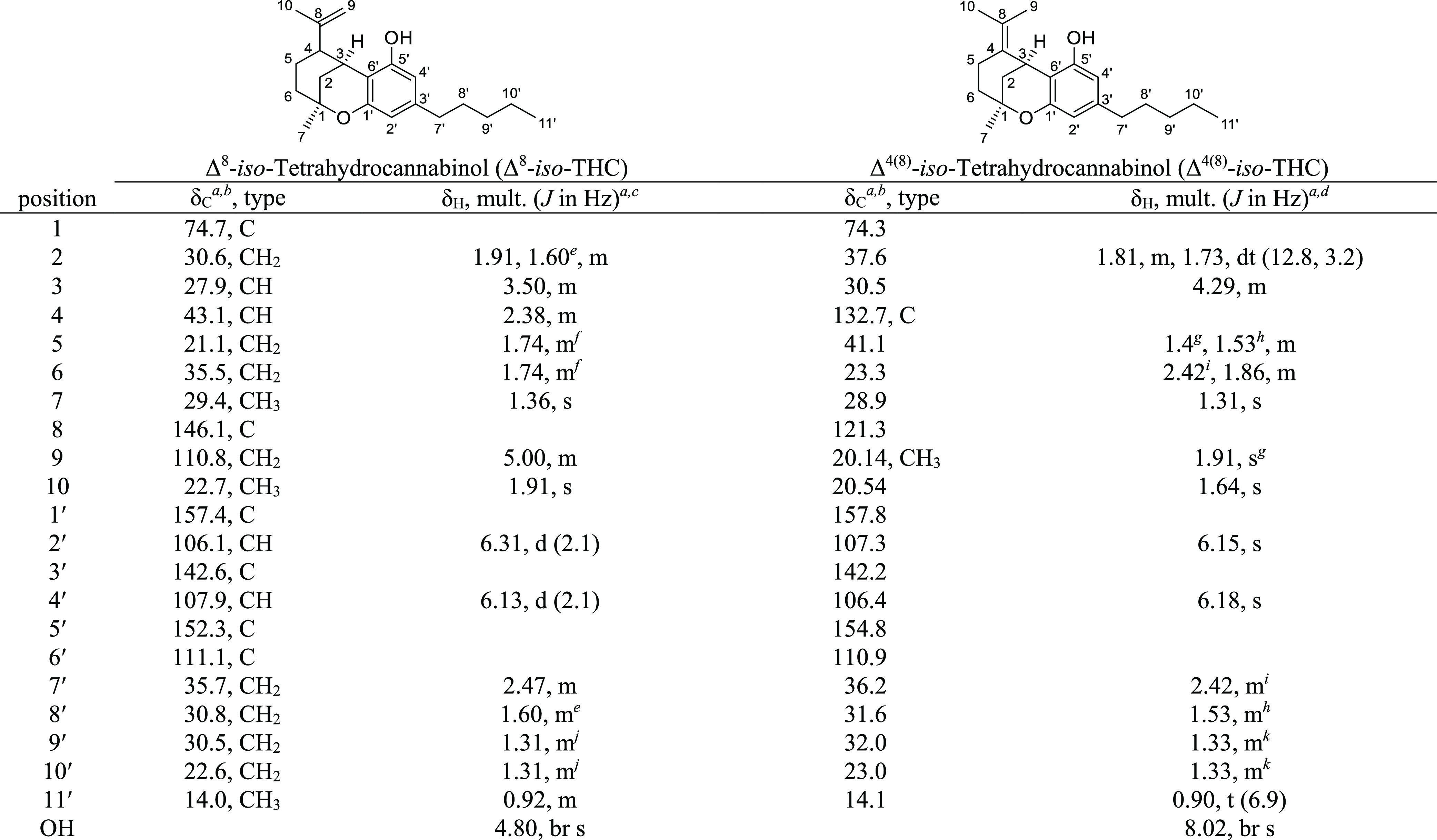
NMR Spectroscopy Data (300 MHz, Acetone-*d*_6_) of Δ^8^-*iso*-THC and (400 MHz, CDCl_3_) of Δ^4(8)^-*iso*-THC

a Chemical shifts (in ppm) were determined with reference to TMS.

b Spectra recorded at 101 MHz.

c Spectra recorded at 300 MHz.

d Spectra recorded at 400 MHz.

e–k Chemical shifts bearing the same symbol overlap.

#### Δ^4(8)^-*iso*-THC

[α]_D_^20^ −236 (*c* 1, CHCl_3_); [HPLC ASCENTIS C_18_; RT Δ^4(8)^*-iso*-THC = 29.62 min]; ^1^H and ^13^C NMR data see Table 6; HRMS (ESI) *m*/*z* [M + Na]^+^ 337.2133 (calcd. for C_21_H_30_O_2_Na, 337.2138).

### BF_3_·OEt_2_-Catalyzed Reactions (Table 1)

Reactions were performed as specified in the general procedure for Lewis acids.

Conditions (Table 1, entry 1): CBD (315 mg, 1 mmol); solvent, anhydrous CH_2_Cl_2_ (5 mL); *T* = −10 °C; BF_3_·OEt_2_ (151 μL, 1.2 mmol); reaction time, 4 h. Yields: Δ^9^-THC, 138 mg (44%); Δ^8^-THC, 4 mg (1%); Δ^8^-*iso*-THC, 11 mg (3%).

Conditions (Table 1, entry 2): CBD (315 mg, 1 mmol); solvent, anhydrous CH_2_Cl_2_ (5 mL); *T* = 0 °C; BF_3_·OEt_2_ (151 μL, 1.2 mmol); reaction time, 6 h. Yields: Δ^9^-THC, 5 mg (2%); Δ^8^-THC, 164 mg (52%).

Conditions (Table 1, entry 3): CBD (315 mg, 1 mmol); solvent, anhydrous CH_2_Cl_2_ (5 mL); *T* = −78 to −30 °C; BF_3_·OEt_2_ (151 μL, 1.2 mmol); reaction time, 48 h. Yields: Δ^9^-THC, 32 mg (10%); Δ^8^-THC, 35 mg (11%); Δ^8^-*iso*-THC, 16 mg (5%).

Conditions (Table 1, entry 4): CBD (156 mg, 0.5 mmol); solvent, anhydrous toluene (2.5 mL); *T* = −10 °C; BF_3_·OEt_2_ (76 μL, 0.6 mmol); reaction time, 3 h. Yields: Δ^9^-THC, 64 mg (41%); Δ^8^-THC, 3 mg (2%); Δ^8^-*iso*-THC, 45 mg (29%).

Conditions (Table 1, entry 5): CBD (316 mg, 1 mmol); solvent, anhydrous toluene (5 mL); *T* = 0 °C; BF_3_·OEt_2_ (151 μL, 1.2 mmol); reaction time, 6 h. Yields: Δ^8^-THC, 115 mg (36%); Δ^4(8)^-*iso*-THC, 83 mg (26%).

Conditions (Table 1, entry 7): CBD (315 mg, 1 mmol); solvent, anhydrous MeCN (5 mL); *T* = −10 °C; BF_3_·OEt_2_ (151 μL, 1.2 mmol); reaction time, 6 h. Yields: Δ^8^-THC, 16 mg (5%); Δ^8^-*iso*-THC, 95 mg (30%); Δ^4(8)^-*iso*-THC, 17 mg (5%).

### TMSOTf-Catalyzed Reactions (Table 1)

Reactions were performed as specified in the general procedure for Lewis acids.

Conditions (Table 1, entry 8): CBD (315 mg, 1 mmol); solvent, anhydrous CH_2_Cl_2_ (5 mL); *T* = −10 °C; TMSOTf (217 μL, 1.2 mmol); reaction time, 6 h. Yields: Δ^8^-THC, 293 mg (93%).

Conditions (Table 1, entry 9): CBD (80 mg, 0.25 mmol); solvent, anhydrous toluene (1.25 mL); *T* = −10 °C; TMSOTf (91 μL, 0.5 mmol); reaction time, 6 h. Yields: Δ^9^-THC, 10 mg (12%); Δ^8^-THC, 61 mg (75%).

### In(OTf)_3_-Catalyzed Reactions (Table 1)

Reactions were performed as specified in the general procedure for Lewis acids.

Conditions (Table 1, entry 10): CBD (317 mg, 1 mmol); solvent, anhydrous CH_2_Cl_2_ (5 mL); *T* = −10 °C; In(OTf)_3_ (675 mg, 1.2 mmol); reaction time, 6 h. Yields: Δ^9^-THC, 165 mg (52%); Δ^*8*^-THC, 18 mg (6%); Δ^8^-*iso*-THC, 12 mg (4%).

Conditions (Table 1, entry 11): CBD (317 mg, 1 mmol); solvent, anhydrous CH_2_Cl_2_ (5 mL); *T* = 0 °C to RT; In(OTf)_3_ (58 mg, 0.1 mmol); reaction time, 48 h. Yields: Δ^8^-THC, 228 mg (72%).

Conditions (Table 1, entry 13): CBD (156 mg, 0.5 mmol); solvent, anhydrous toluene (2.5 mL); *T* = 0 °C; In(OTf)_3_ (563 mg, 1 mmol); reaction time, 24 h. Yields: Δ^8^-THC, 153 mg (98%).

### TiCl_4_-Catalyzed Reaction (Table 1)

The reaction was performed as specified in the general procedure for Lewis acids.

Conditions (Table 1, entry 15): CBD (315 mg, 1 mmol); solvent, anhydrous CH_2_Cl_2_ (5 mL); *T* = −10 °C; TiCl_4_ (167 μL, 1.2 mmol); reaction time, 6 h. Yields: CBD, 38 mg (12%); Δ^9^-THC, 108 mg (34%); Δ^8^-THC, 27 mg (9%).

### HCl-Catalyzed Reaction (Table 2)

Reaction was performed as specified in the general procedure for protic acids.

Conditions (Table 2, entry 1): CBD (156 mg, 0.5 mmol); solvent, H_2_O (1.6 mL); *T* = RT; HCl 37% (1.6 mL); reaction time, 72 h. Yields: Δ^8^-THC, 89 mg (57%).

### pTSA·H_2_O-Catalyzed Reactions (Table 2)

Reactions were performed as specified in the general procedure for protic acids.

Conditions (Table 2, entry 2): CBD (154 mg, 0.5 mmol); solvent, anhydrous CH_2_Cl_2_ (2.5 mL); *T* = RT; pTSA·H_2_O (189 mg, 1 mmol); reaction time, 36 h. Yields: Δ^8^-THC, 145 mg (94%).

Conditions (Table 2, entry 3): CBD (155 mg, 0.5 mmol); solvent, *n*-hexane (2.5 mL); *T* = RT; pTSA·H_2_O (190 mg, 1 mmol); reaction time, 36 h. Yields: Δ^9^-THC, 20 mg (13%); Δ^8^-THC, 102 mg (66%); Δ^8^-*iso*-THC, 20 mg (13%).

Conditions (Table 2, entry 5): CBD (318 mg, 1 mmol); solvent, anhydrous toluene (5 mL); *T* = RT; pTSA·H_2_O (386 mg, 2 mmol); reaction time, 48 h. Yields: Δ^9^-THC, 262 mg (82%); Δ^8^-THC, 34 mg (11%).

Conditions (Table 2, entry 6): CBD (79 mg, 0.25 mmol); solvent, anhydrous toluene (1.25 mL); *T* = RT; pTSA·H_2_O (6 mg, 0.025 mmol); reaction time, 96 h. Yields: Δ^9^-THC, 7 mg (9%); Δ^8^-THC, 70 mg (89%).

### CSA-Catalyzed Reaction (Table 2)

The reaction was performed as specified in the general procedure for protic acids.

Conditions (Table 2, entry 7): CBD (79 mg, 0.25 mmol); solvent, anhydrous toluene (1.25 mL); *T* = RT; CSA (117 mg, 0.5 mmol); reaction time, 96 h. Yields: CBD, 28 mg (36%); Δ^9^-THC, 48 mg (61%).

### H_2_SO_4_-Catalyzed Reactions (Table 2)

Reactions were performed as specified in the general procedure for protic acids.

Conditions (Table 2, entry 8): CBD (315 mg, 1 mmol); solvent, anhydrous CH_2_Cl_2_ (5 mL); *T* = 0 °C; H_2_SO_4_, 98% (54 μL, 1 mmol); reaction time, 72 h. Yields: Δ^8^-THC, 16 mg (5%); Δ^8^-*iso*-THC, 13 mg (4%); Δ^4(8)^-*iso*-THC, 37 mg (11%).

### General Procedure for CSA Screening Reactions (Table 3)

The procedure is the same as described in the general procedure for Lewis acids, but the reactions were quenched by diluting with EtOAc and were monitored by TLC (*n*-hexane/EtOAc 7:3, eluted 2 times) developed by cerium molybdate stain. Crudes were analyzed by ^1^H NMR spectroscopy in CDCl_3_ and HPLC to determine the composition. Conditions: CBD (1 equiv); CSA (2 equiv); solvent, toluene (0.2 M) or as specified in Table 3; *T* as specified in Table 3.

## Supporting Information Available

NMR spectra of compounds (PDF)
