# Supplementary material for: Cannabidiol as the Substrate in Acid-Catalyzed Intramolecular Cyclization
Source: J Nat Prod. 2020 Sep 29;83(10):2894–901. doi: 10.1021/acs.jnatprod.0c00436 (PMC8011986; doi:10.1021/acs.jnatprod.0c00436)
Supplement: Supplementary file 1 — np0c00436_si_001.pdf [file np0c00436_si_001.pdf]

Cannabidiol as the Substrate in Acid-Catalysed Intramolecular Cyclization

Paola Marzullo,[†] *Francesca Foschi,*[†,‡] *Davide Andrea Coppini, Fabiola Fanchini, Lucia Magnani, Selina Rusconi,*[§] *Marcello Luzzani,*[§] *Daniele Passarella**

Dipartimento di Chimica, Università degli Studi di Milano, 20133 Milan, Italy

‡Dipartimento di Scienza e Alta Tecnologia, Università degli Studi dell'Insubria, 22100 Como, Italy; §LINNEA SA, 6595 Riazzino (TI), Switzerland

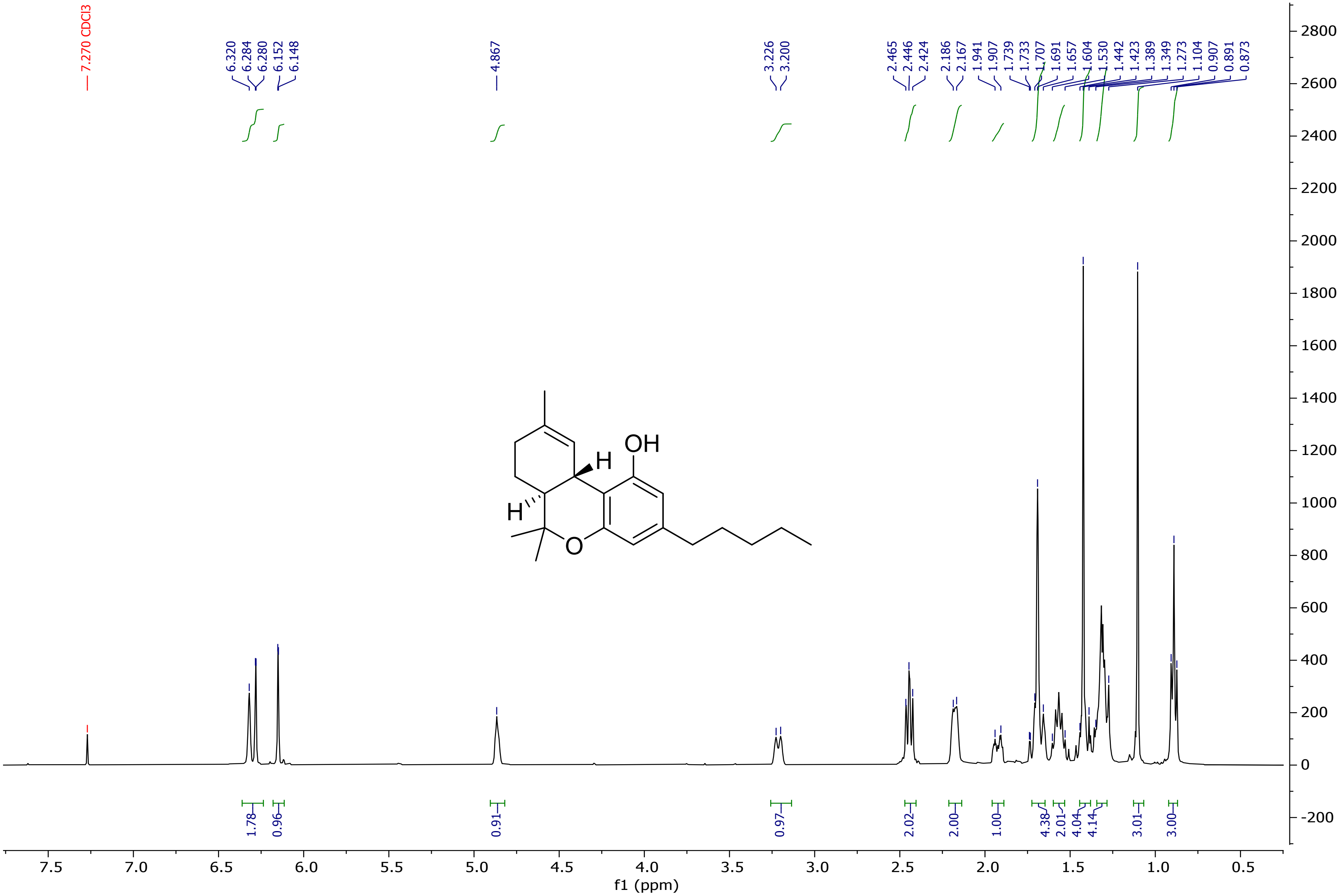

Figure S1. ^{1}H NMR Spectrum (400 MHz, $CDCl_3$) of Δ^9-THC

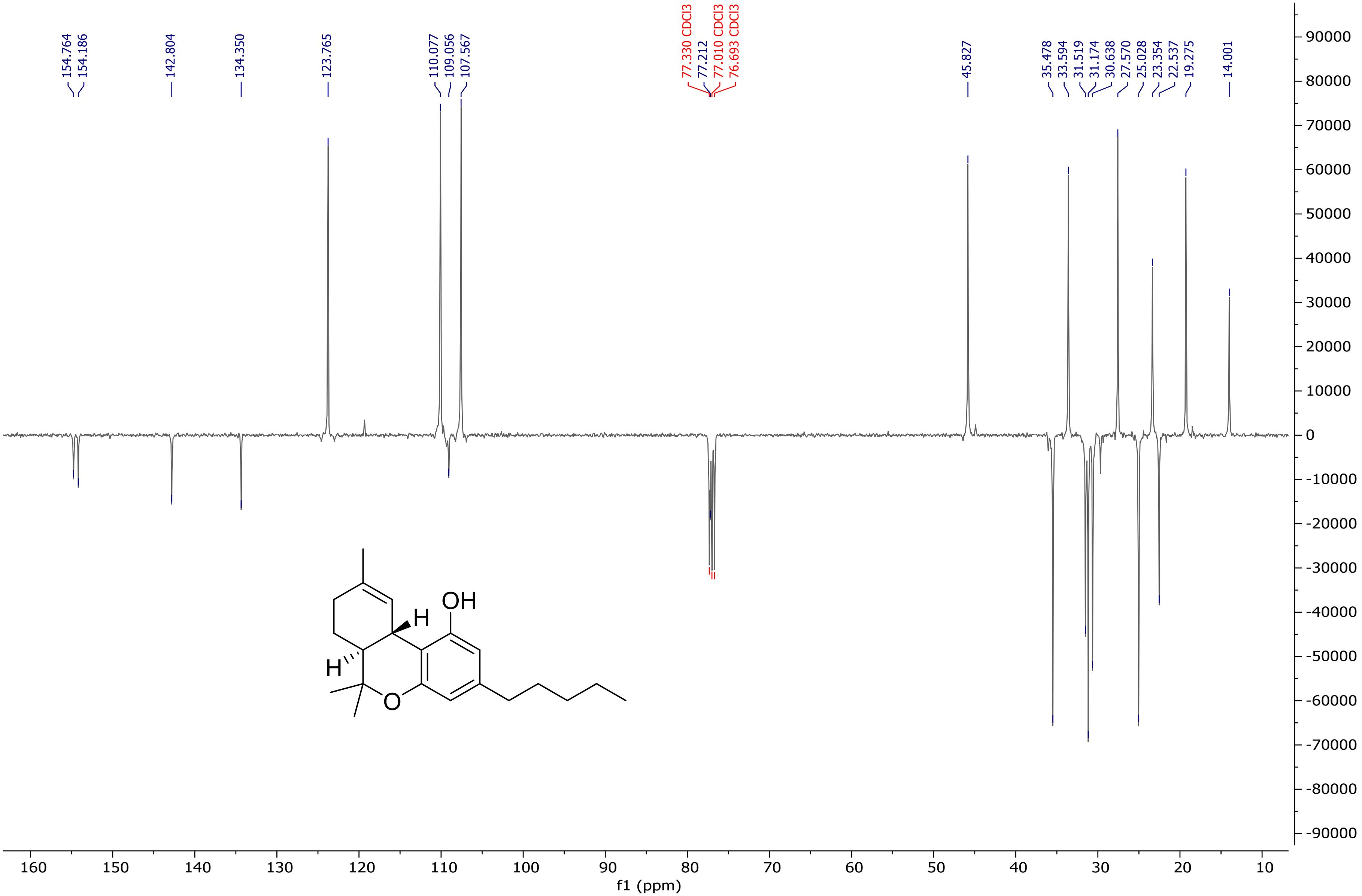

Figure S2. ^{13}C NMR APT Spectrum (101 MHz, $CDCl_3$) of Δ^9-THC

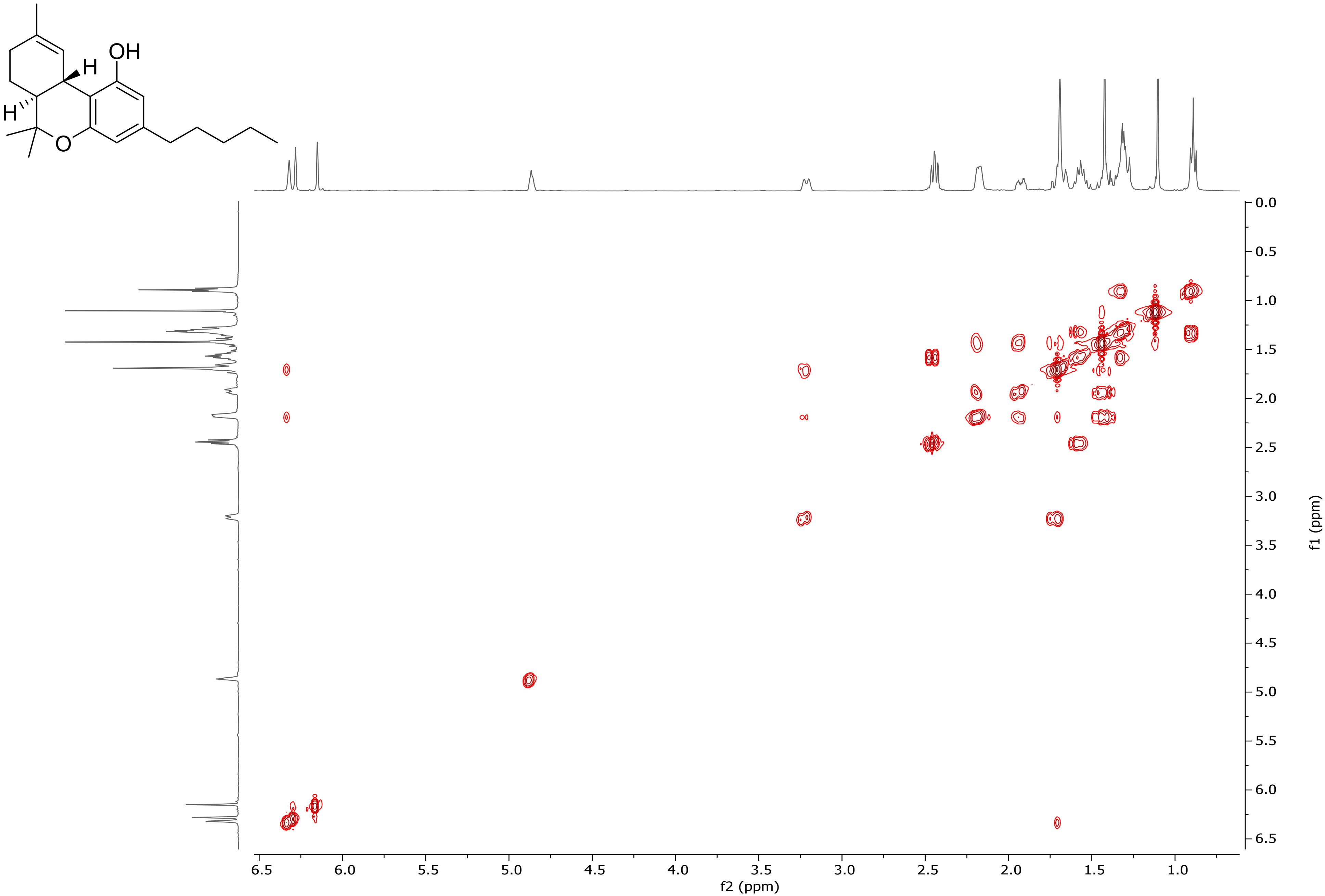
OH
H
H
O
6.5 6.0 5.5 5.0 4.5 4.0 3.5 3.0 2.5 2.0 1.5 1.0
f2 (ppm)
0.0 0.5 1.0 1.5 2.0 2.5 3.0 3.5 4.0 4.5 5.0 5.5 6.0 6.5
f1 (ppm)

Figure S3. ^{1}H COSY Spectrum in $CDCl_3$ of Δ^9-THC

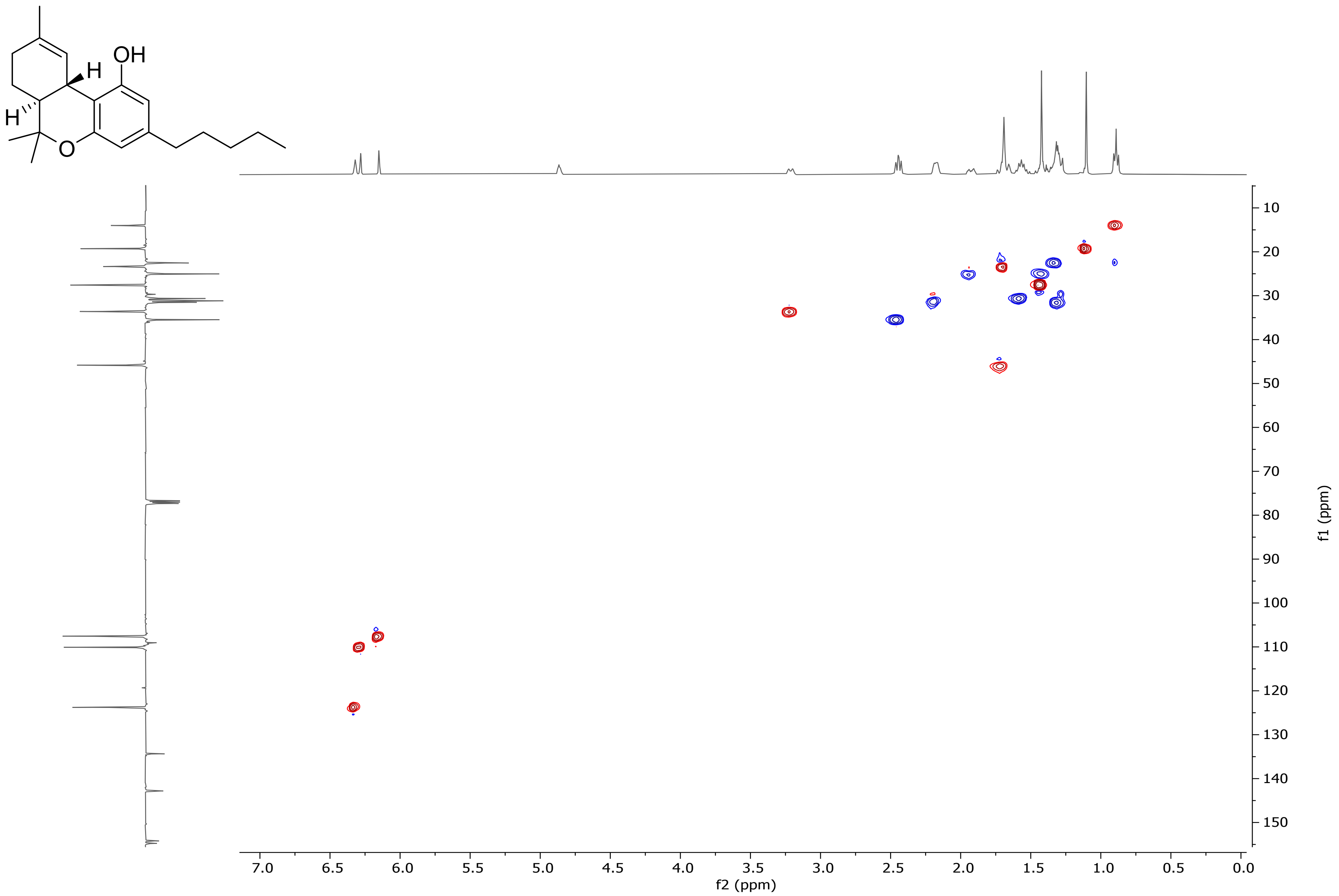

Figure S4. HSQC Spectrum in $CDCl_3$ of Δ^9-THC

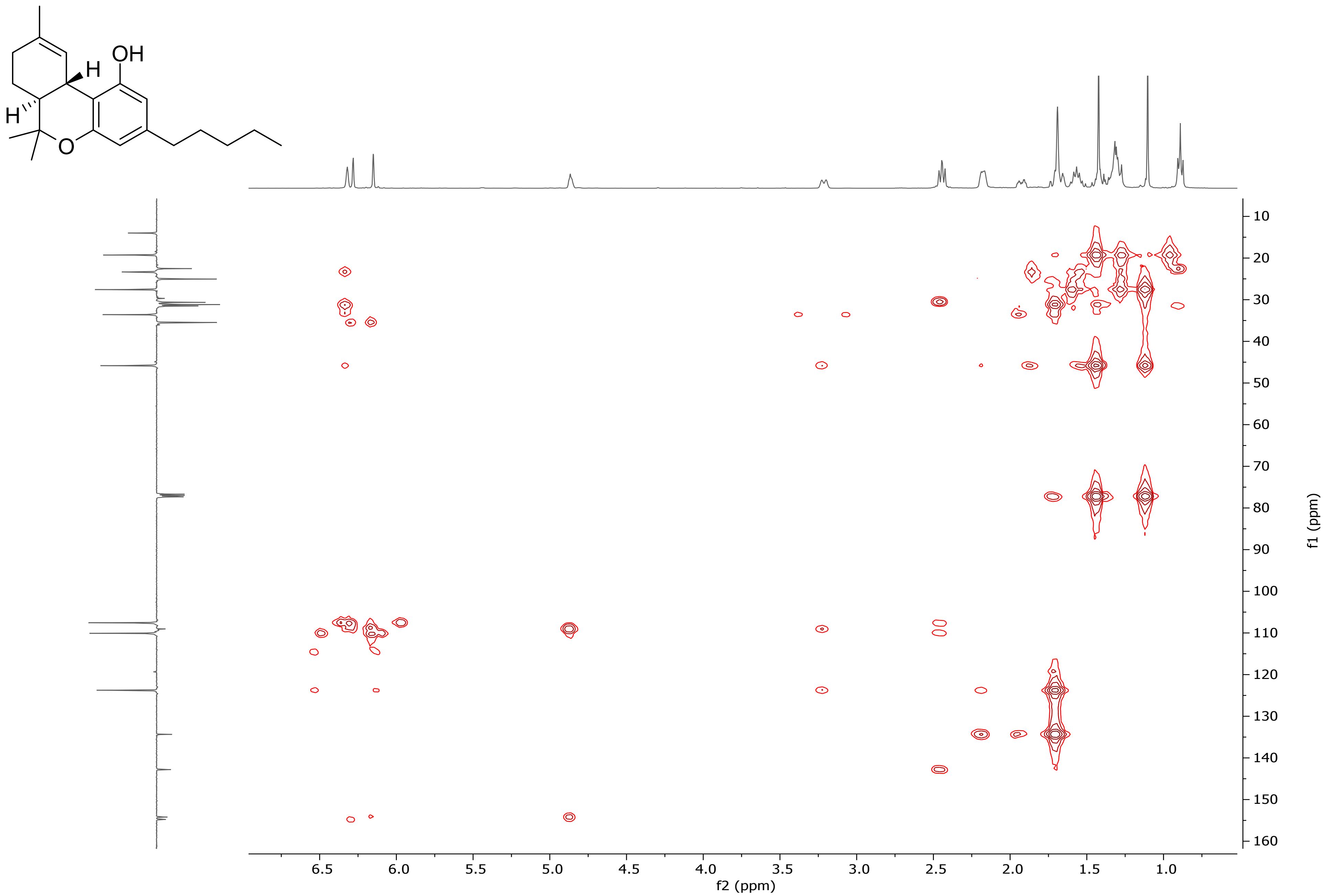

Figure S5. HMBC Spectrum in $CDCl_3$ of Δ^9-THC

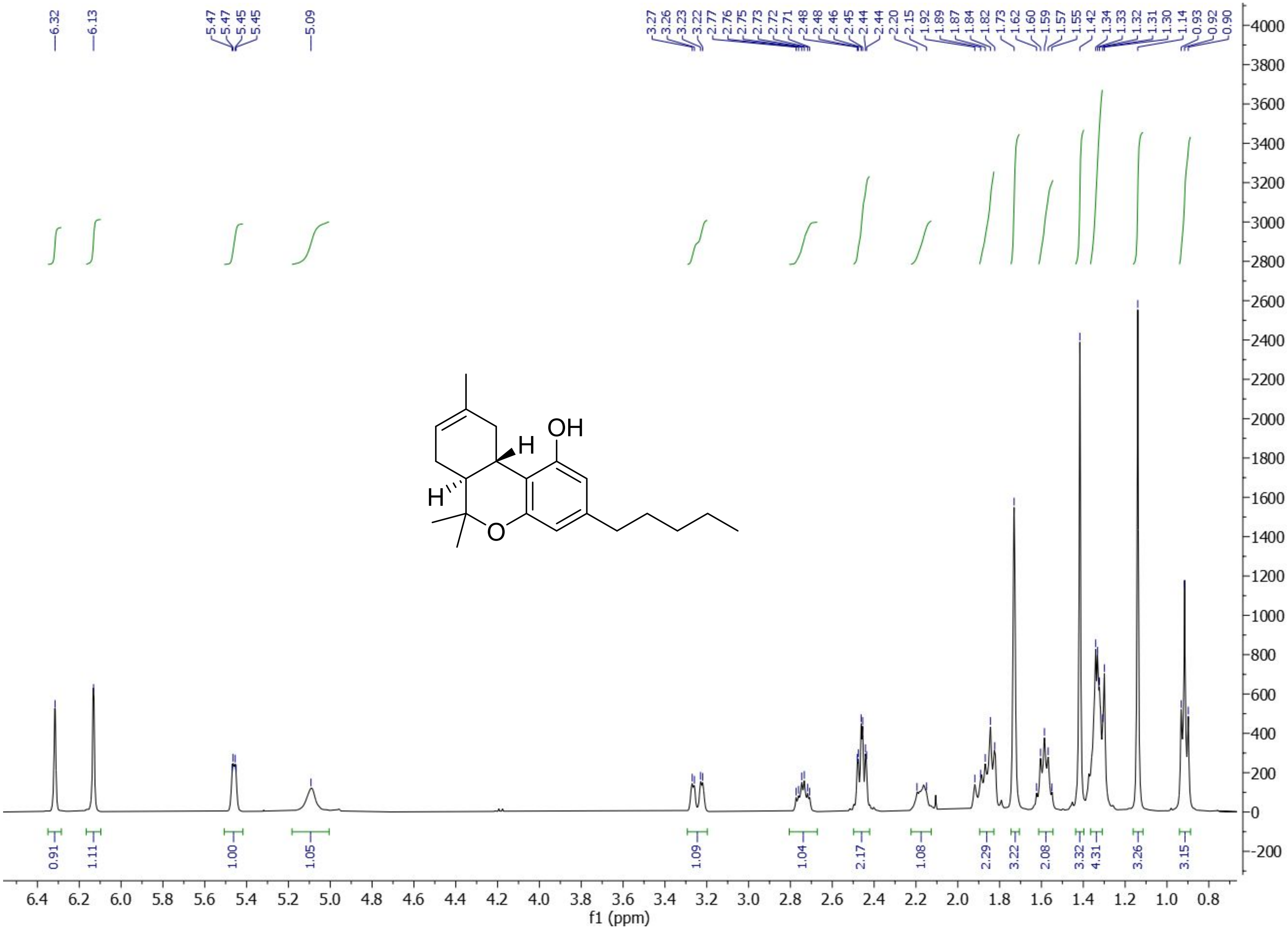

Figure S6. ^{1}H NMR Spectrum (400 MHz, $CDCl_3$) of Δ^8-THC

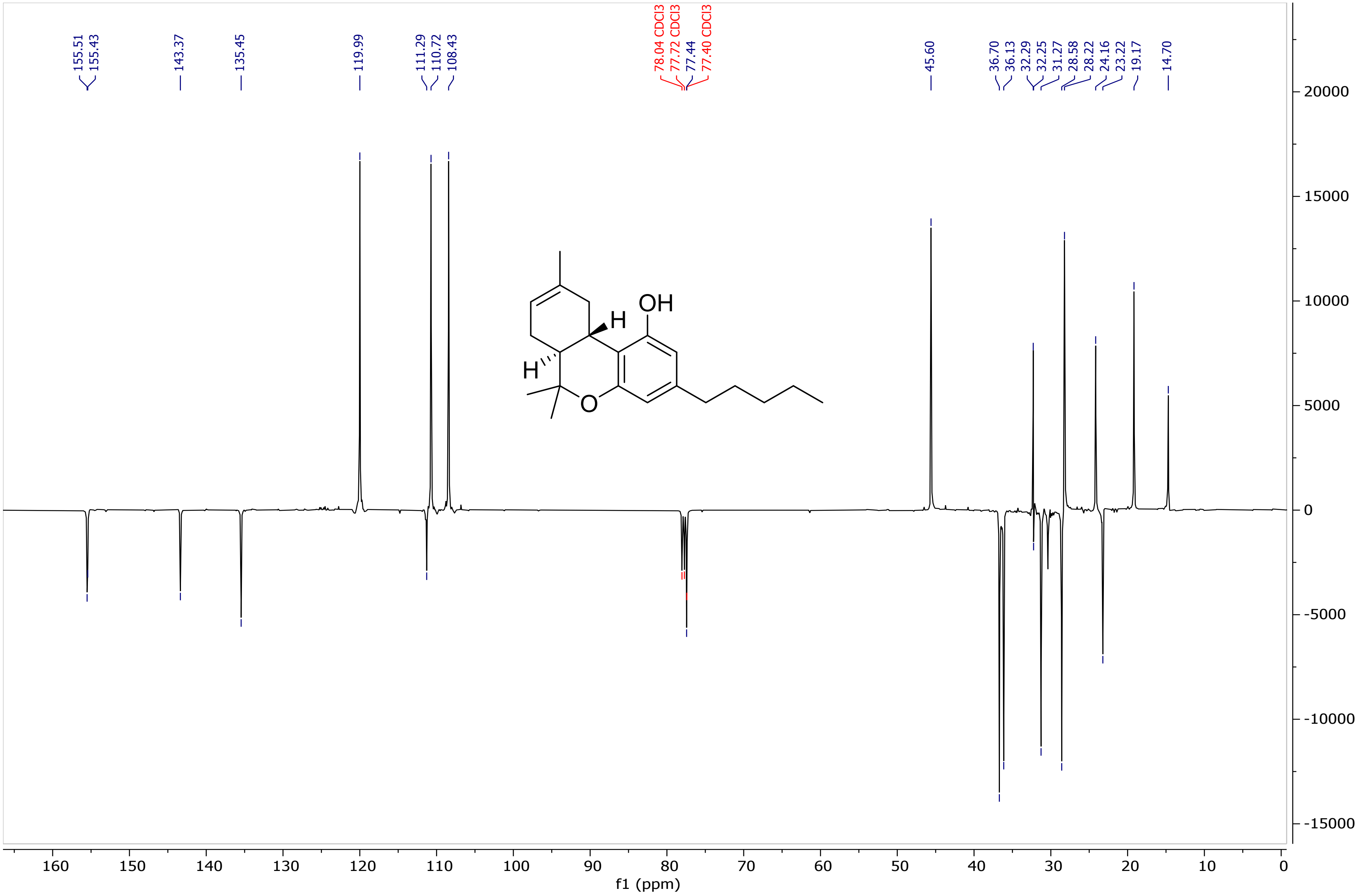

Figure S7. ^{13}C NMR APT Spectrum (101 MHz, $CDCl_3$) of Δ^8-THC

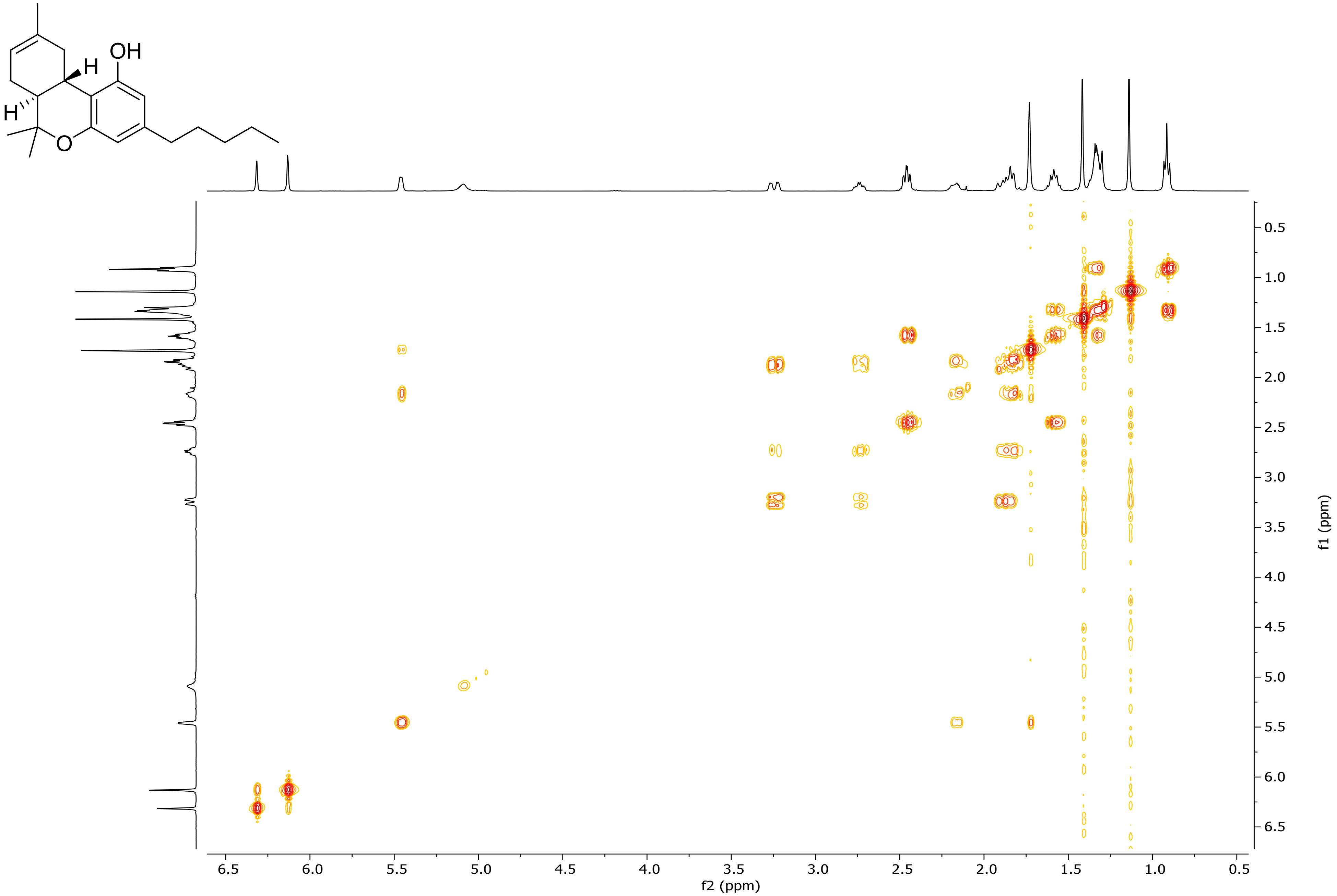

Figure S8. ^{1}H COSY Spectrum in $CDCl_3$ of Δ^8-THC

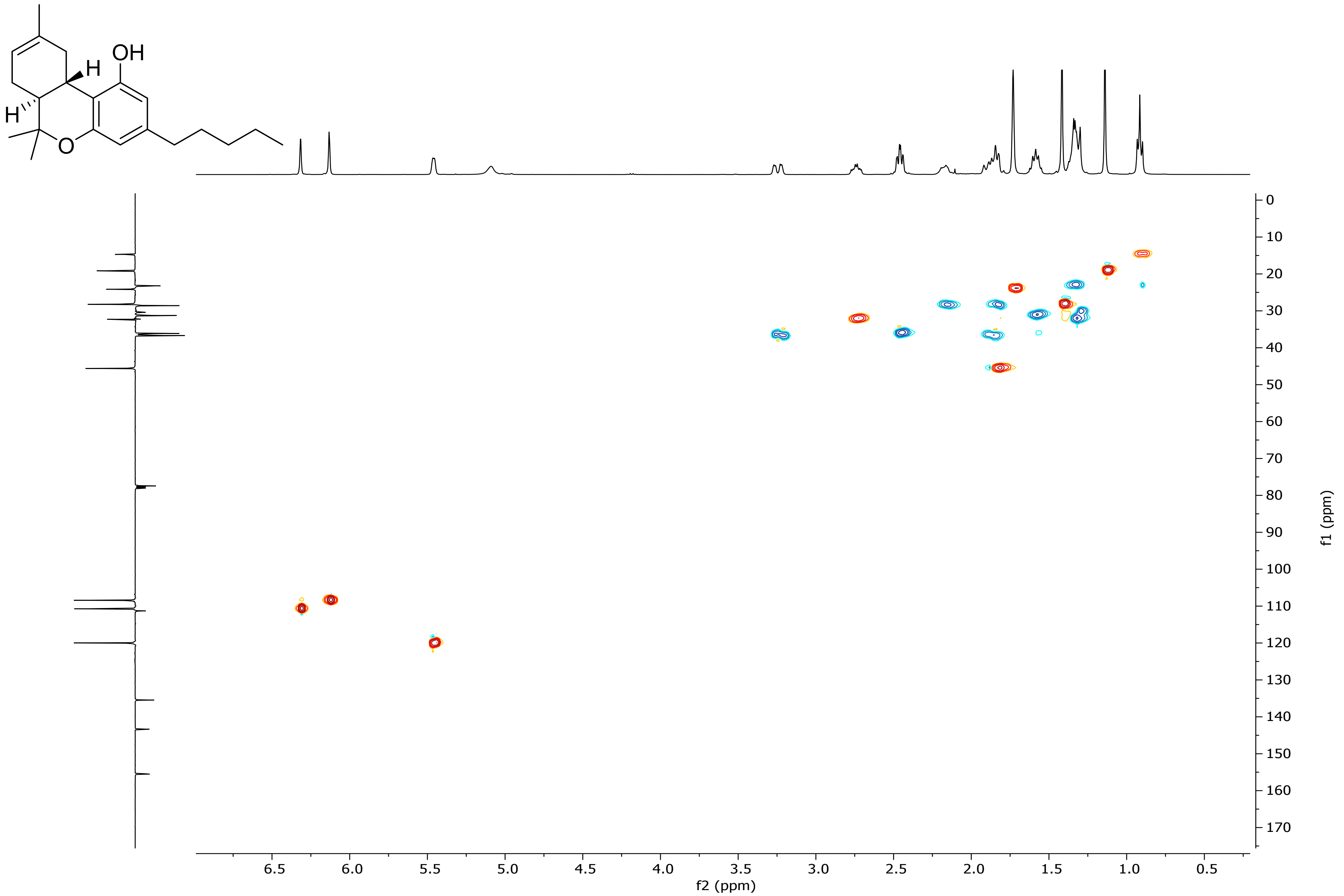

Figure S9. HSQC Spectrum in $CDCl_3$ of Δ^8-THC

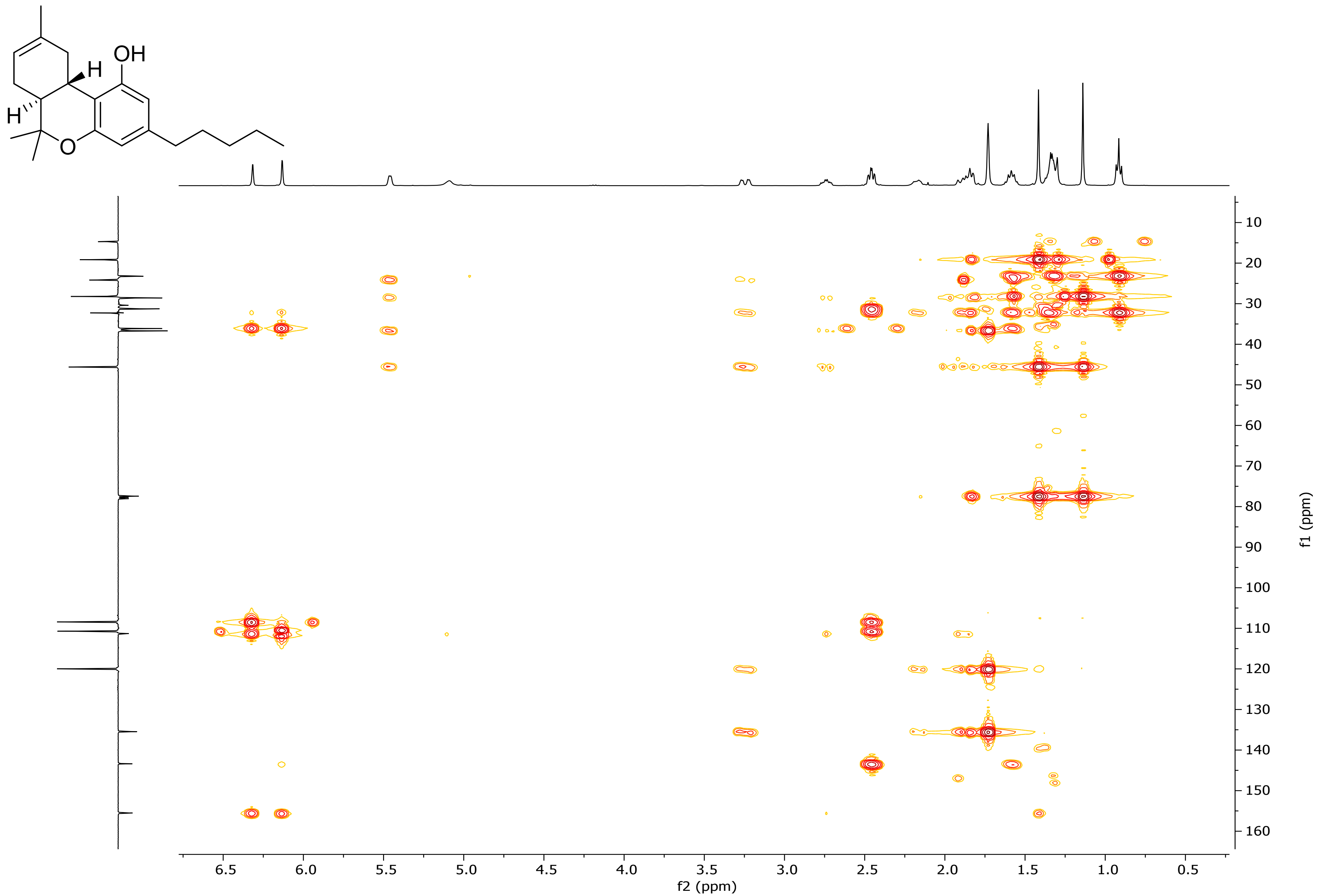

Figure S10. HMBC Spectrum in $CDCl_3$ of Δ^8-THC

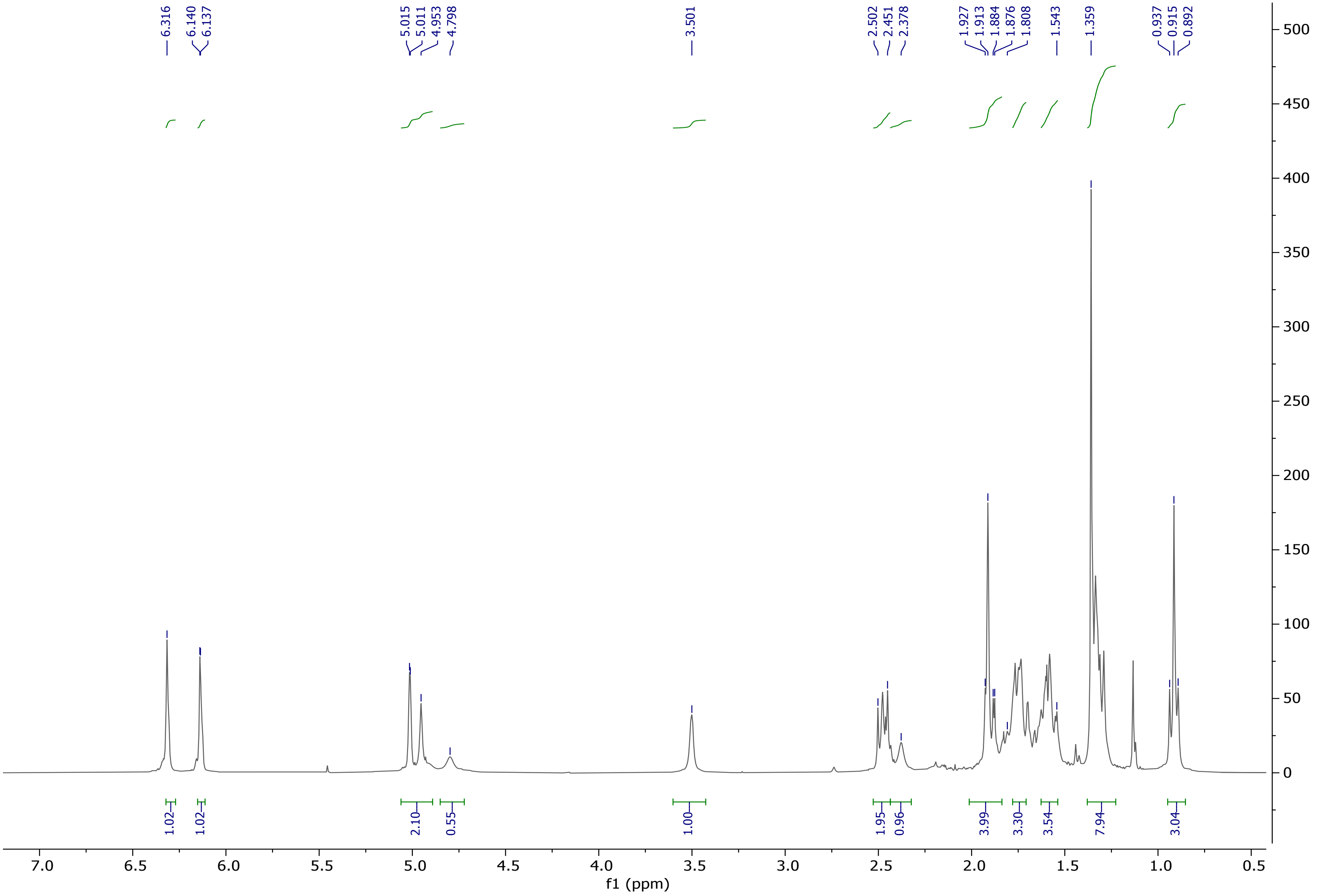

6.316
6.140
6.137
5.015
5.011
4.953
4.798
3.501
2.502
2.451
2.378
1.927
1.913
1.884
1.876
1.808
1.543
1.359
0.937
0.915
0.892
1.02
1.02
2.10
0.55
1.00
1.95
0.96
3.99
3.30
3.54
7.94
3.04
f1 (ppm)

Figure S11. ^{1}H NMR Spectrum (300 MHz, $CDCl_3$) of Δ^8-*iso*-THC

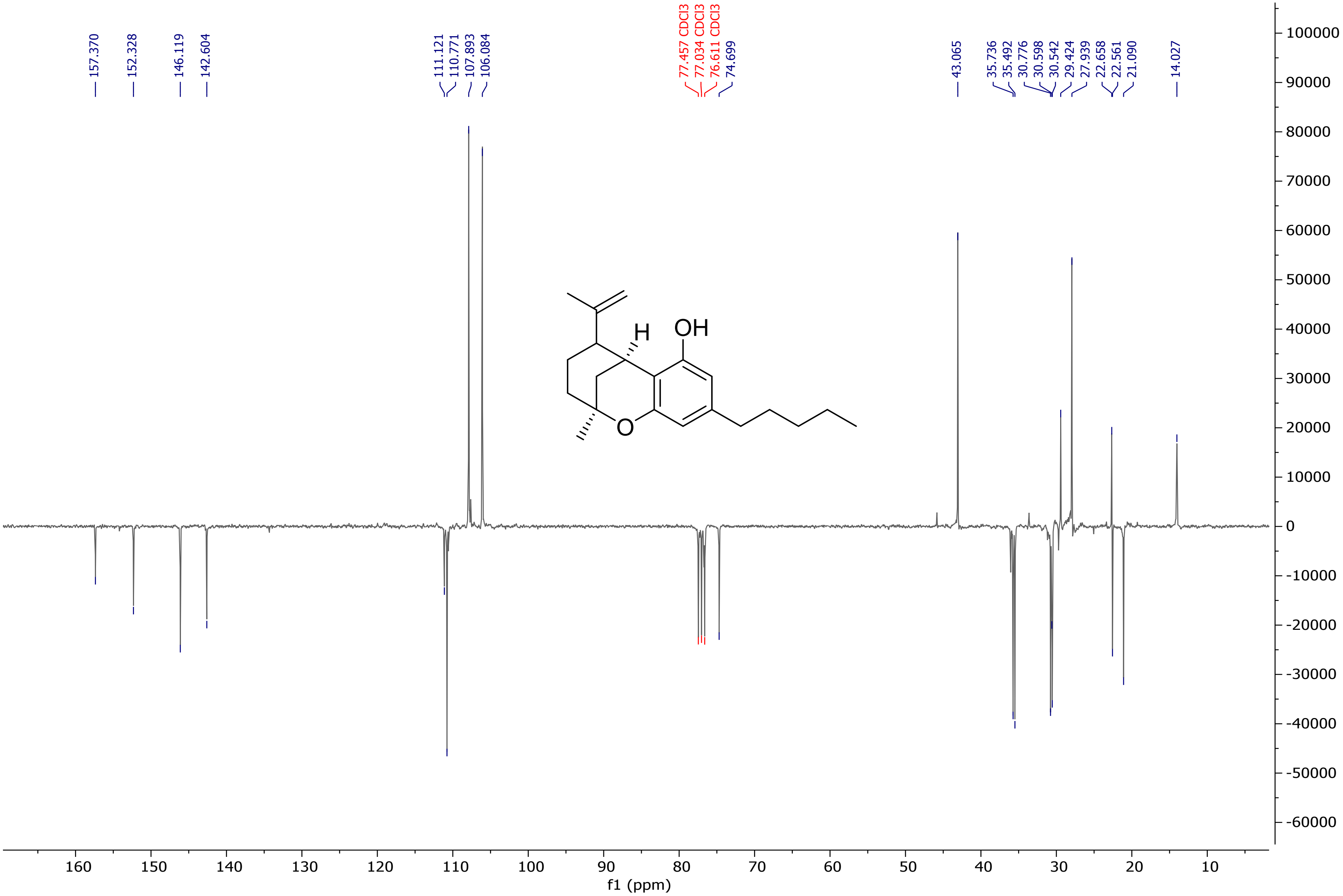
157.370
152.328
146.119
142.604
111.121
110.771
107.893
106.084
77.457 CDCl3
77.034 CDCl3
76.611 CDCl3
74.699
43.065
35.736
35.492
30.776
30.598
30.542
29.424
27.939
22.658
22.561
21.090
14.027
OH
H
O
f1 (ppm)

Figure S12. ^{13}C NMR APT Spectrum (101 MHz, $CDCl_3$) of Δ^8-*iso*-THC

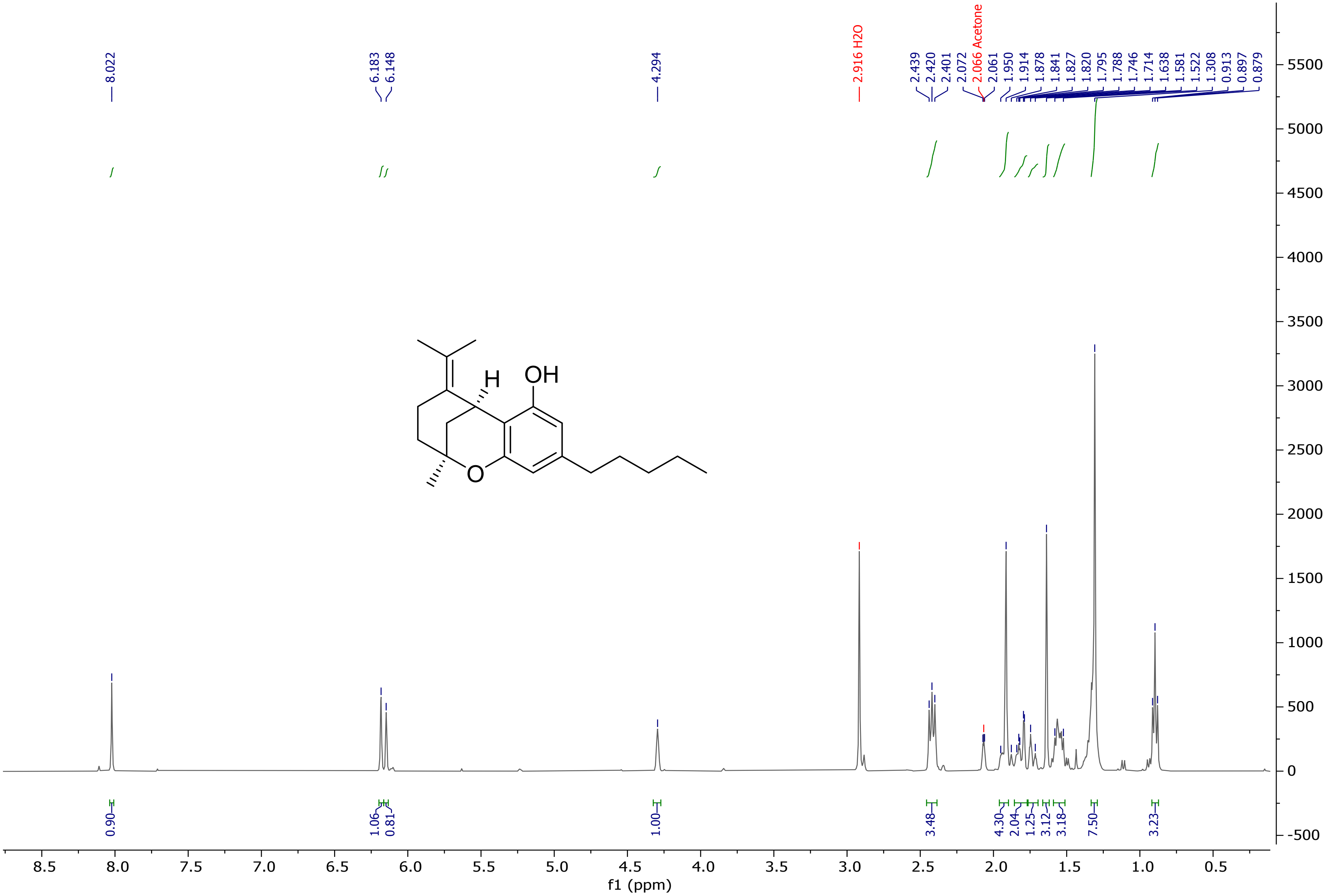
8.022
6.183
6.148
4.294
2.916 H2O
2.439
2.420
2.401
2.072
2.066 Acetone
2.061
1.950
1.914
1.878
1.841
1.827
1.820
1.795
1.788
1.746
1.714
1.638
1.581
1.522
1.308
0.913
0.897
0.879
H
OH
O
0.90
1.06
0.81
1.00
3.48
4.30
2.04
1.25
3.12
3.18
7.50
3.23
f1 (ppm)

Figure S13. ^{1}H NMR Spectrum (400 MHz, acetone-d_6) of $\Delta^{4(8)}$-*iso*-THC

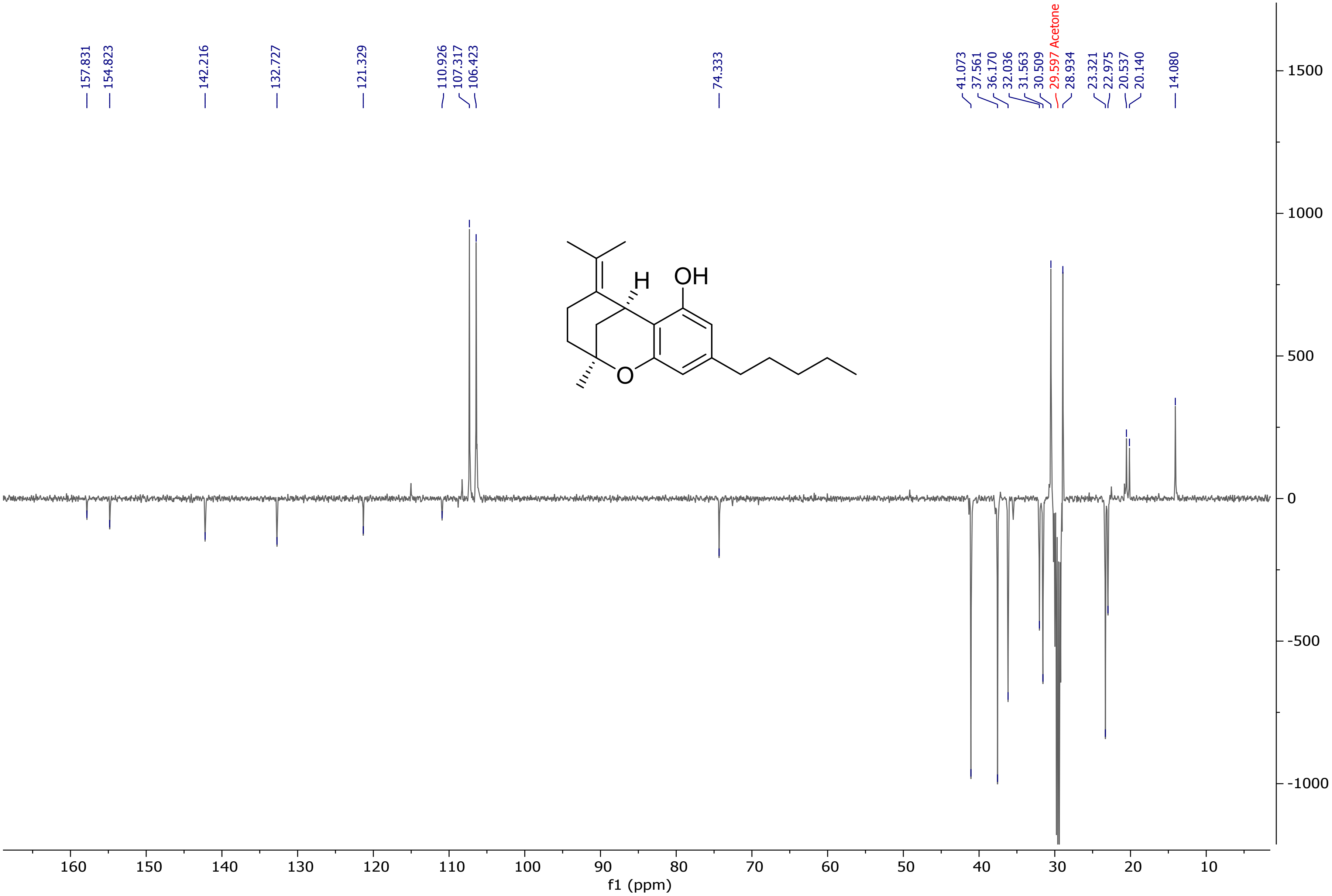
157.831
154.823
142.216
132.727
121.329
110.926
107.317
106.423
74.333
41.073
37.561
36.170
32.036
31.563
30.509
29.597 Acetone
28.934
23.321
22.975
20.537
20.140
14.080
H
OH
O
1500
1000
500
0
-500
-1000
160
150
140
130
120
110
100
90
80
70
60
50
40
30
20
10
f1 (ppm)

Figure S14. ^{13}C NMR APT Spectrum (101 MHz, acetone-d_6) of $\Delta^{4(8)}$-*iso*-THC

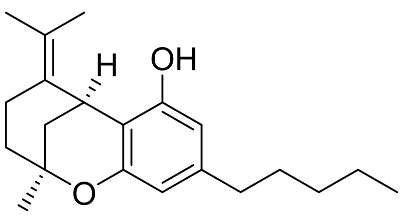
H
OH
O

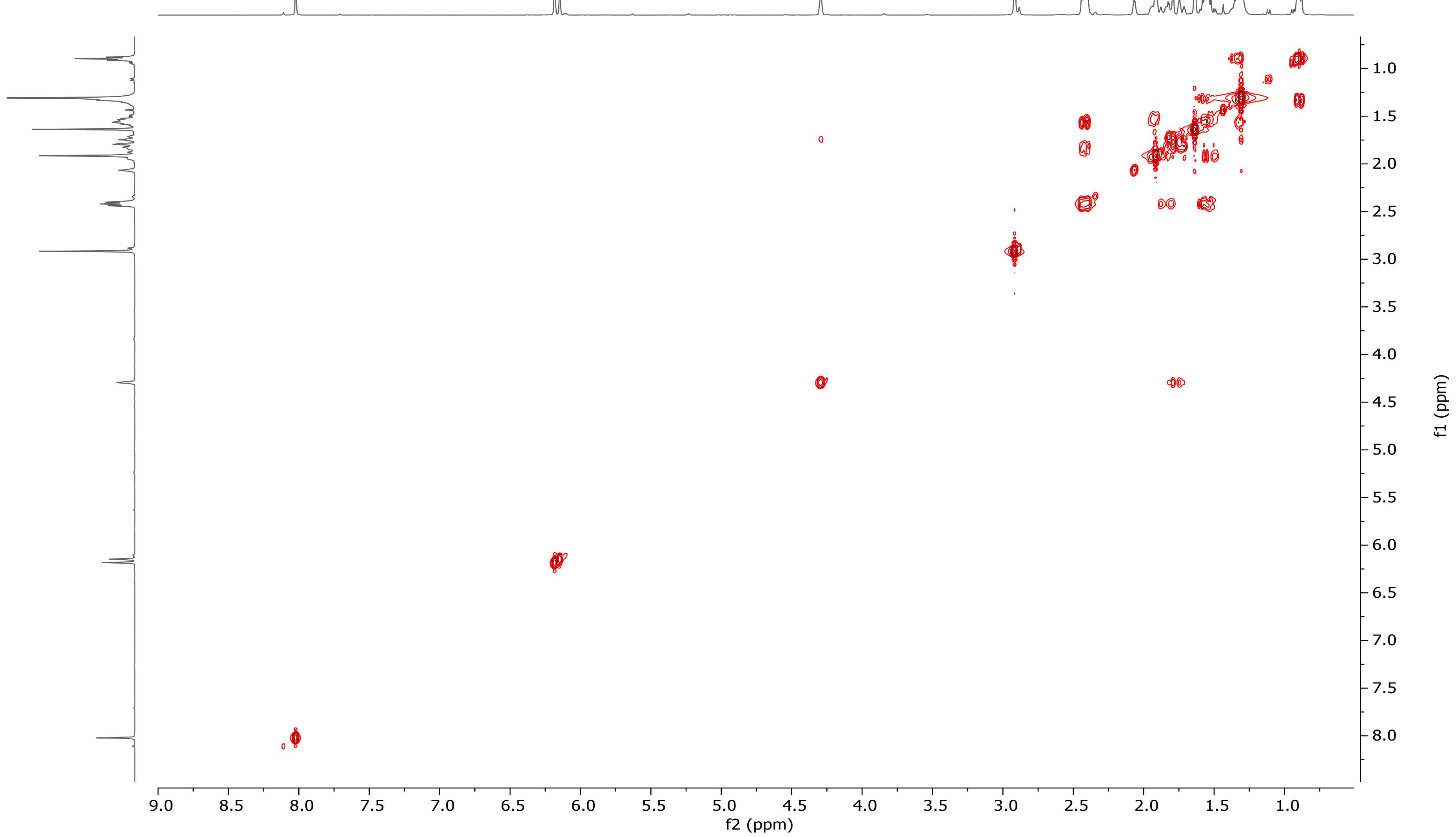
f2 (ppm)
f1 (ppm)
9.0
8.5
8.0
7.5
7.0
6.5
6.0
5.5
5.0
4.5
4.0
3.5
3.0
2.5
2.0
1.5
1.0

Figure S15. ^{1}H COSY Spectrum in acetone-d_6 of $\Delta^{4(8)}$-*iso*-THC

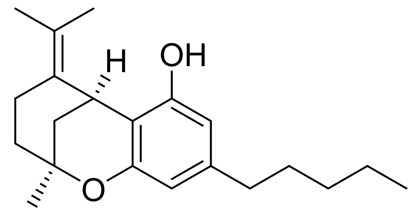

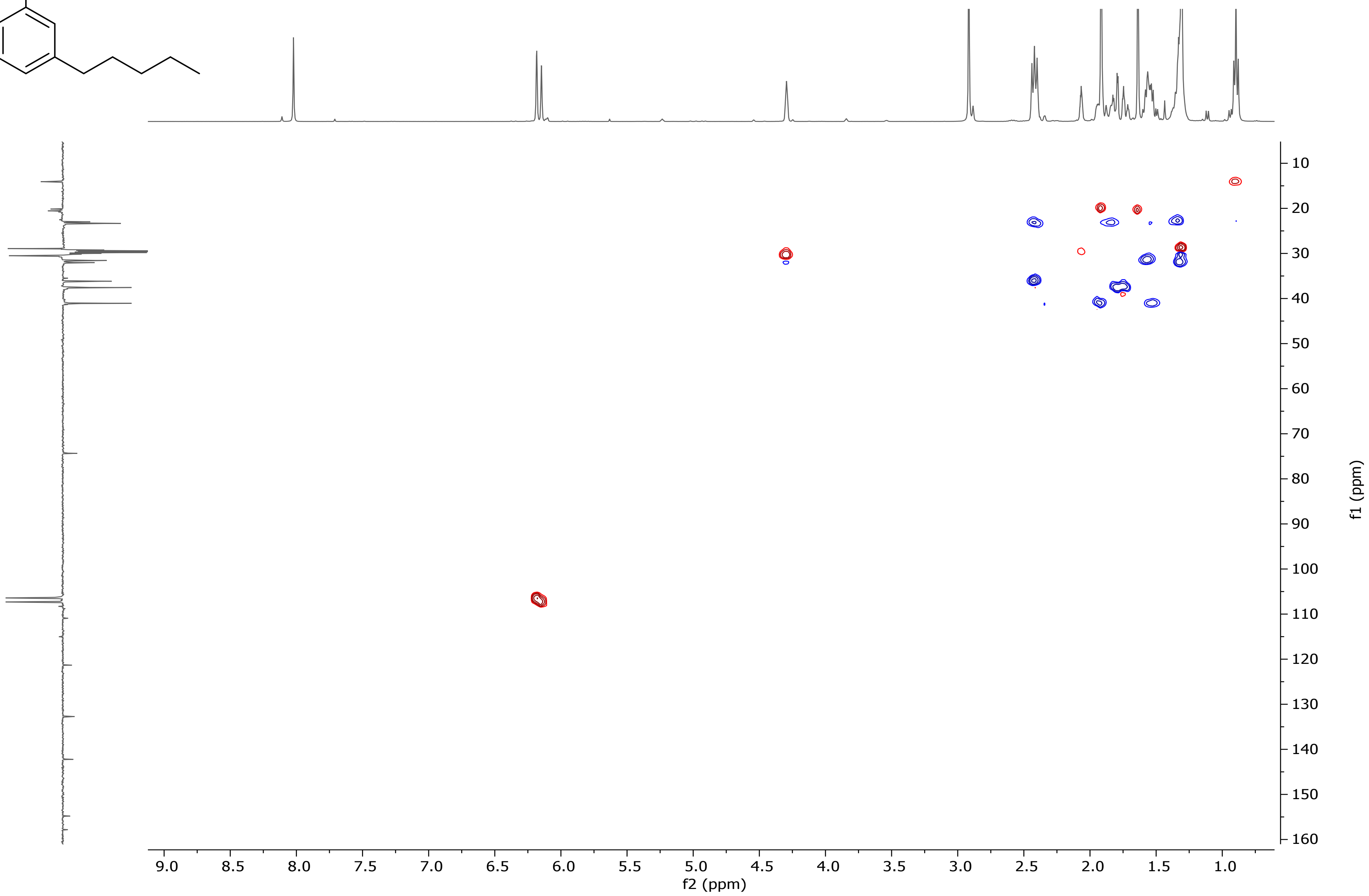

10
20
30
40
50
60
70
80
90
100
110
120
130
140
150
160
f1 (ppm)
9.0
8.5
8.0
7.5
7.0
6.5
6.0
5.5
5.0
4.5
4.0
3.5
3.0
2.5
2.0
1.5
1.0
f2 (ppm)

Figure S16. HSQC Spectrum in acetone-d_6 of $\Delta^{4(8)}$-*iso*-THC

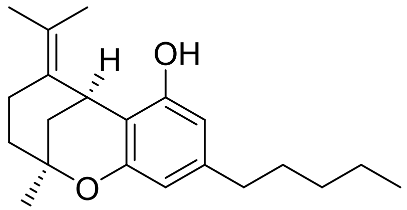
H
OH
O

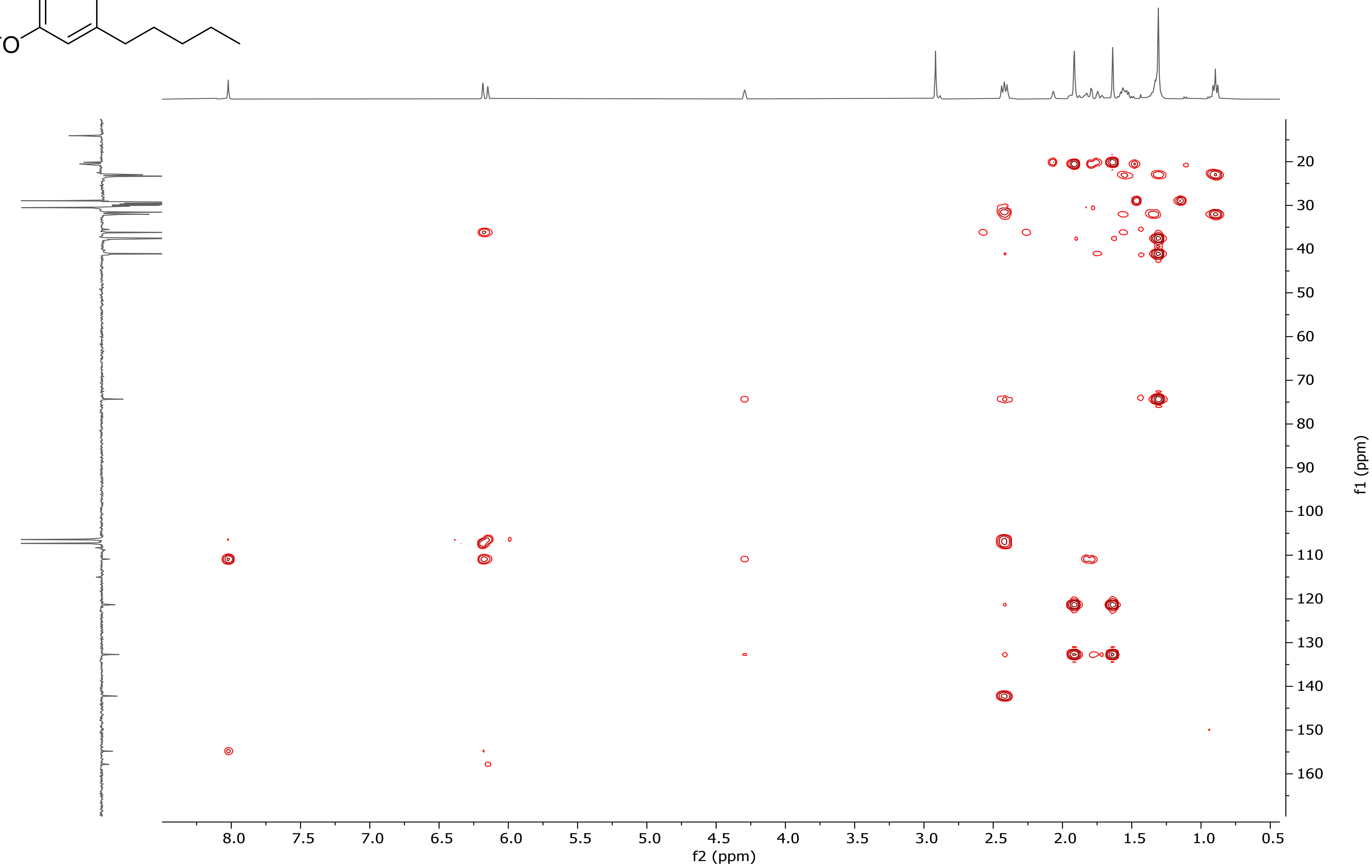
f1 (ppm)
f2 (ppm)
20
30
40
50
60
70
80
90
100
110
120
130
140
150
160
8.0
7.5
7.0
6.5
6.0
5.5
5.0
4.5
4.0
3.5
3.0
2.5
2.0
1.5
1.0
0.5

Figure S17. HMBC Spectrum in acetone-d_6 of $\Delta^{4(8)}$-*iso*-THC

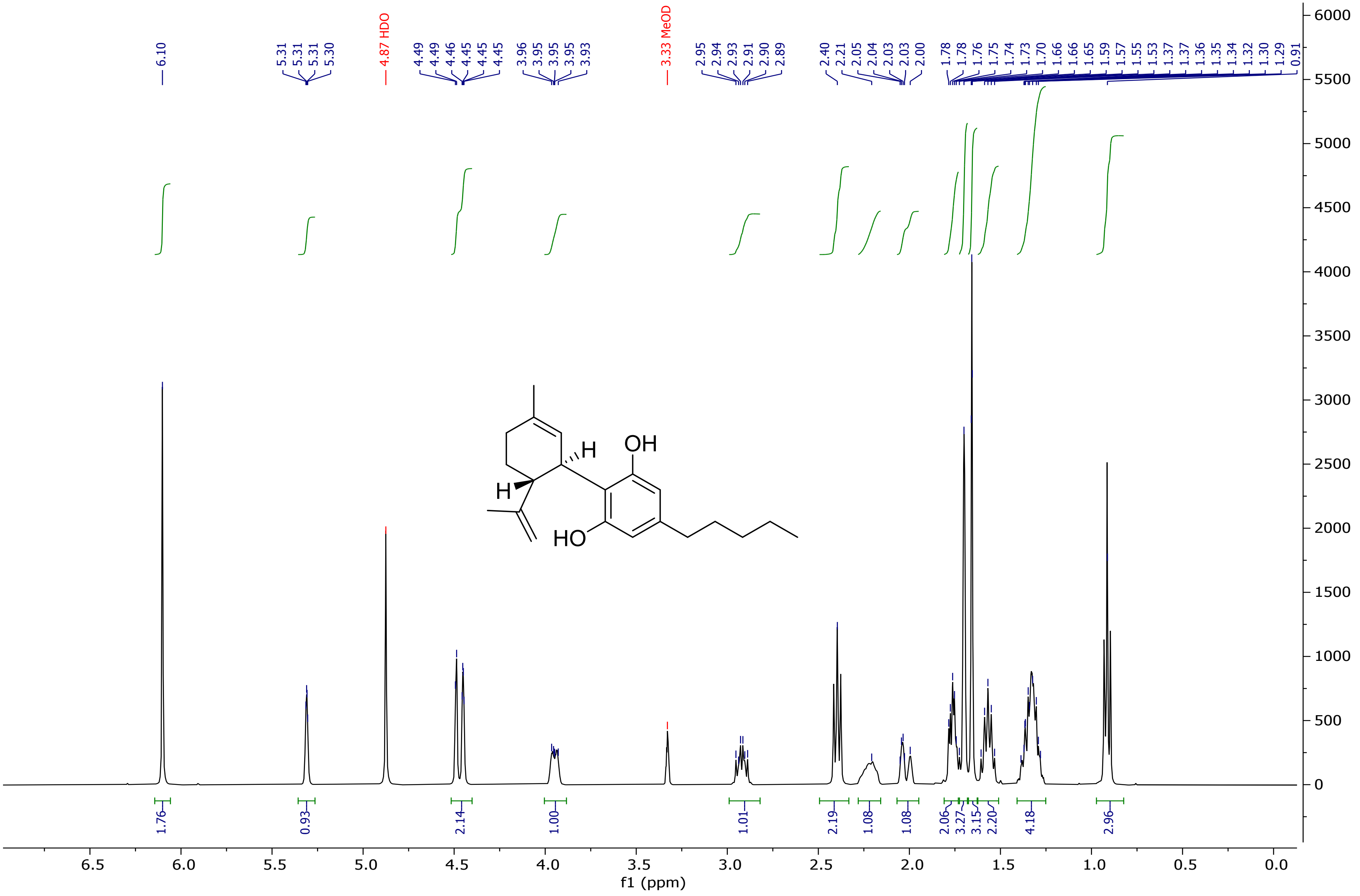
6.10
5.31 5.31 5.31 5.30
4.87 HDO
4.49 4.49 4.46 4.45 4.45 4.45
3.96 3.95 3.95 3.95 3.93
3.33 MeOD
2.95 2.94 2.93 2.91 2.90 2.89
2.40 2.21 2.05 2.04 2.03 2.03 2.00
1.78 1.78 1.76 1.75 1.74 1.73 1.70 1.66 1.66 1.65 1.59 1.57 1.55 1.53 1.37 1.37 1.36 1.35 1.34 1.32 1.30 1.29 0.91
OH
H
H
HO
1.76 0.93 2.14 1.00 1.01 2.19 1.08 1.08 2.06 3.27 3.15 2.20 4.18 2.96
6.5 6.0 5.5 5.0 4.5 4.0 3.5 3.0 2.5 2.0 1.5 1.0 0.5 0.0
f1 (ppm)
6000 5500 5000 4500 4000 3500 3000 2500 2000 1500 1000 500 0

Figure S18. ^{1}H NMR Spectrum (400 MHz, methanol-d_6) of CBD

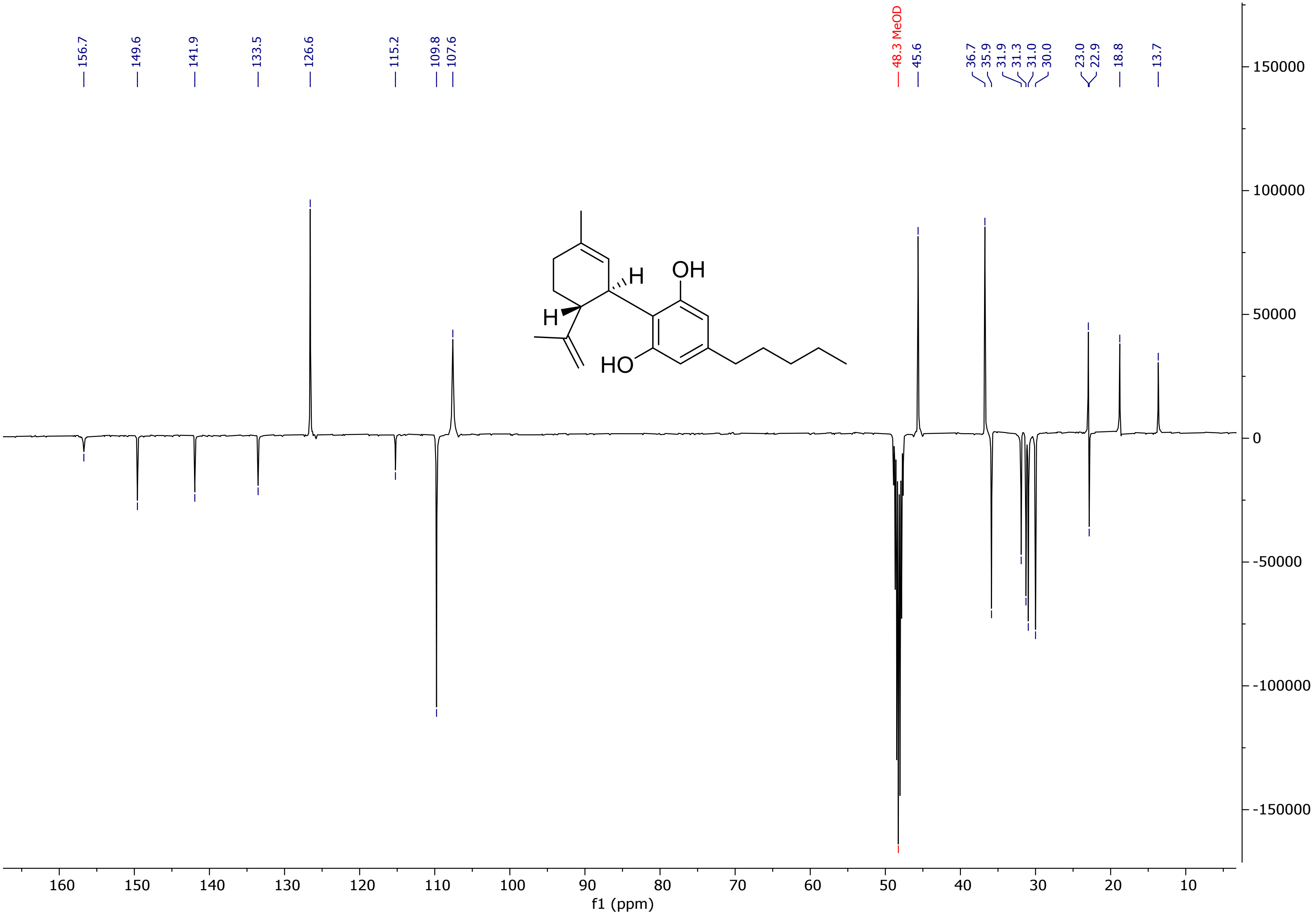
156.7
149.6
141.9
133.5
126.6
115.2
109.8
107.6
48.3 MeOD
45.6
36.7
35.9
31.9
31.3
31.0
30.0
23.0
22.9
18.8
13.7
OH
H
H
HO
150000
100000
50000
0
-50000
-100000
-150000
160
150
140
130
120
110
100
90
80
70
60
50
40
30
20
10
f1 (ppm)

Figure S19. ^{13}C NMR APT Spectrum (101 MHz, methanol-d_4) of CBD

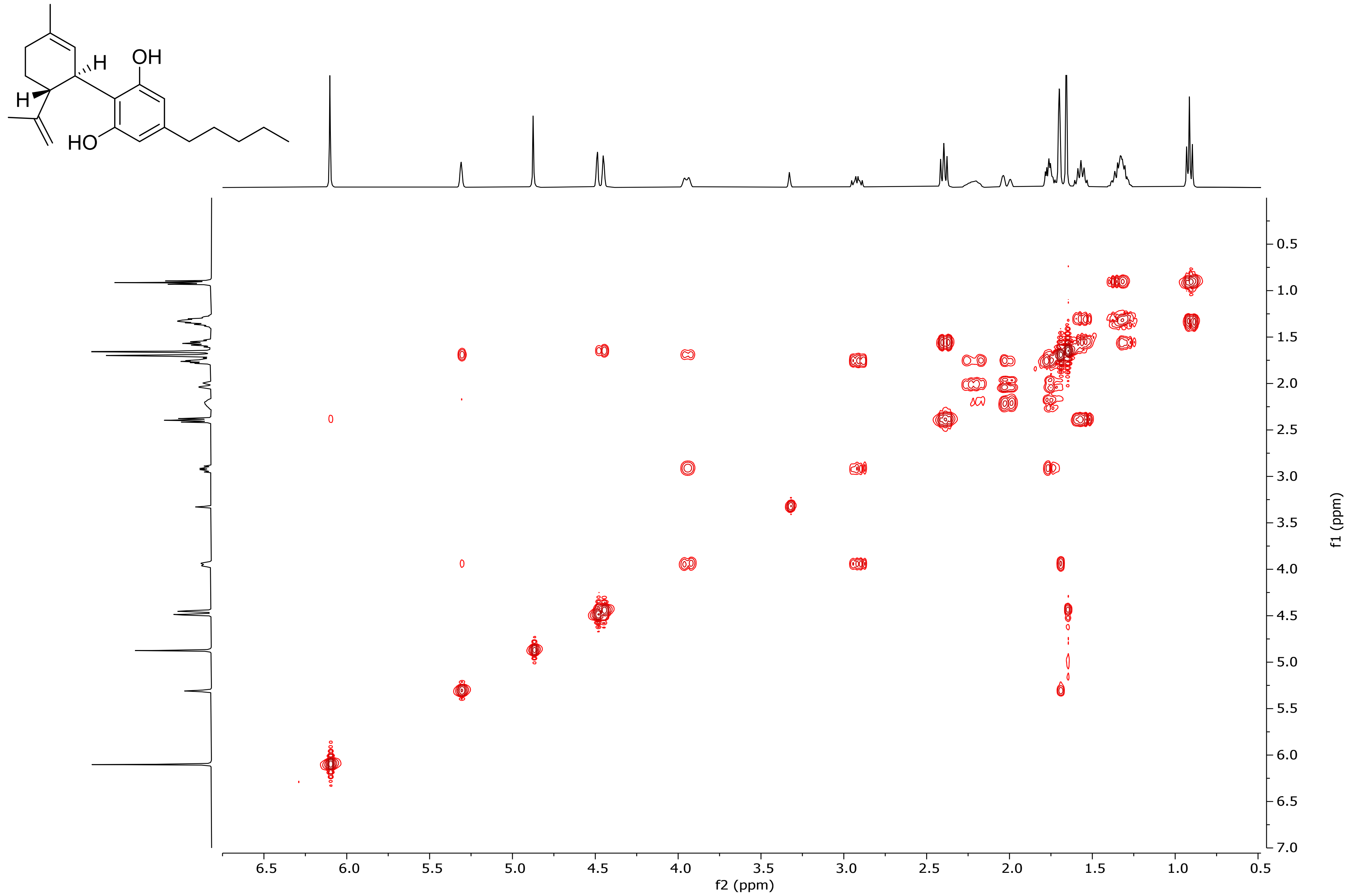
OH
HO
H
H
f2 (ppm)
f1 (ppm)
6.5
6.0
5.5
5.0
4.5
4.0
3.5
3.0
2.5
2.0
1.5
1.0
0.5
7.0

Figure S20. ^1H COSYSpectrum in methanol-d_4 of CBD

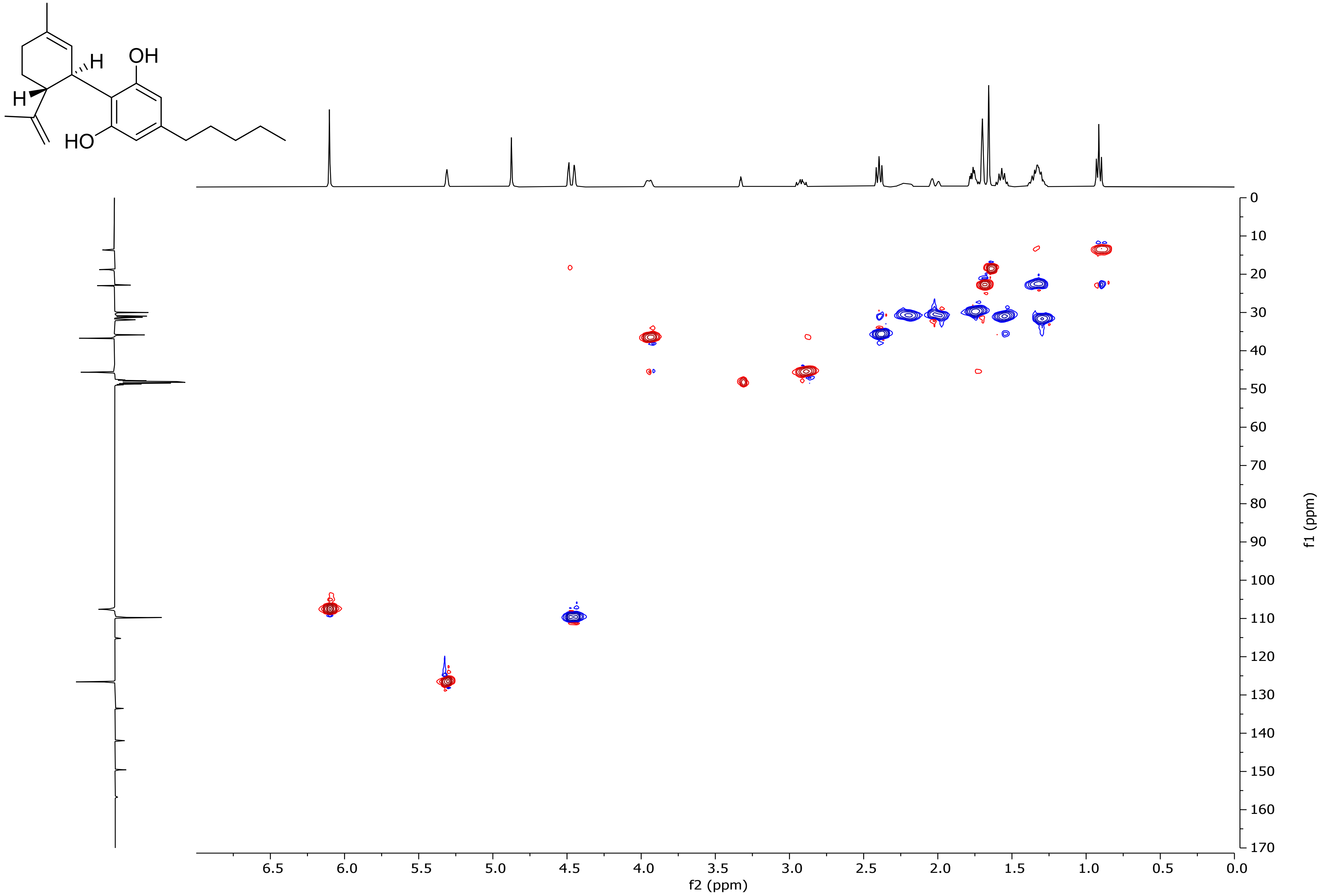
OH
H
H
HO
f2 (ppm)
f1 (ppm)
6.5
6.0
5.5
5.0
4.5
4.0
3.5
3.0
2.5
2.0
1.5
1.0
0.5
0.0
0
10
20
30
40
50
60
70
80
90
100
110
120
130
140
150
160
170

Figure S21. HSQC Spectrum in methanol-d_4 of CBD

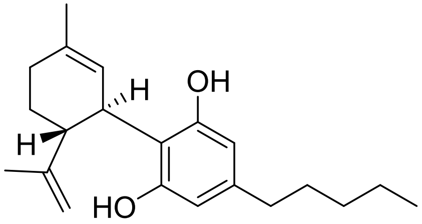
OH
H
H
HO

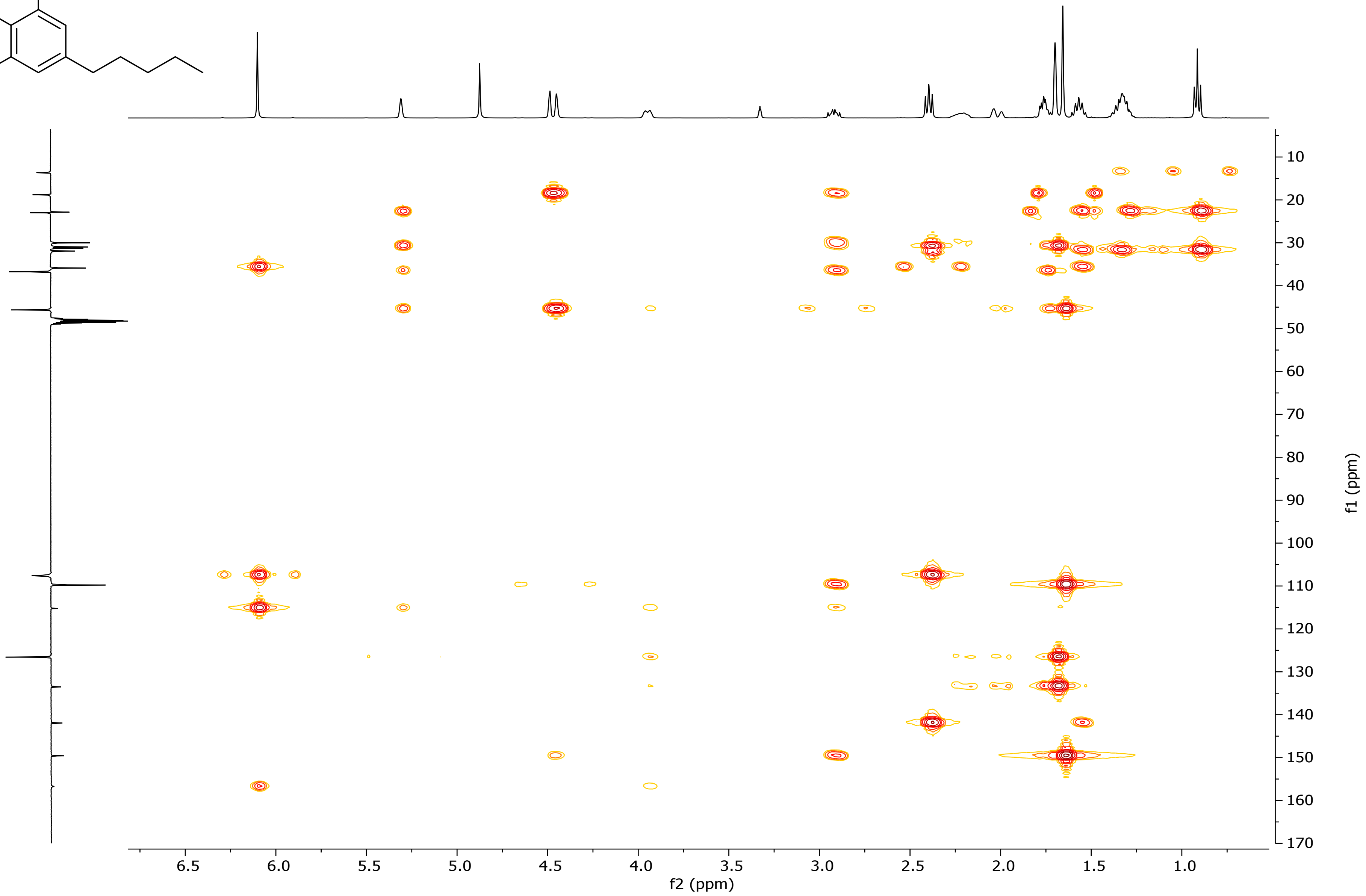
f2 (ppm)
f1 (ppm)
6.5
6.0
5.5
5.0
4.5
4.0
3.5
3.0
2.5
2.0
1.5
1.0
10
20
30
40
50
60
70
80
90
100
110
120
130
140
150
160
170

Figure S22. HMBC Spectrum in methanol-d_4 of CBD
